# A pipeline to characterize spinal cord pathology in neurological disorders combining magnetic resonance microscopy and histopathology

**DOI:** 10.1038/s43856-026-01577-8

**Published:** 2026-04-22

**Authors:** Charidimos Tsagkas, Maxime Donadieu, Roy Sun, Brandon Bujak, Kevin Hu, Camille Rood, Katherine Cameron, Stephen Dodd, Daniel S. Reich, Govind Nair

**Affiliations:** 1 Translational Neuroradiology Section, National Institute of Neurological Disorders and Stroke, National Institutes of Health, Bethesda, MD USA; 2 Translational Imaging in Neurology (ThINk) Basel, Department of Biomedical Engineering, Faculty of Medicine, University Hospital Basel and University of Basel, Basel, MD Switzerland; 3 Neurologic Clinic and Policlinic, Departments of Medicine, University Hospital Basel, Basel, Switzerland; 4 Research Center for Clinical Neuroimmunology and Neuroscience Basel (RC2NB), University Hospital Basel and University of Basel, Basel, Switzerland; 5 Quantitative MRI Core, National Institute of Neurological Disorders and Stroke, National Institutes of Health, Bethesda, MD USA; 6 Section on Instrumentation, National Institute of Mental Health, National Institutes of Health, Bethesda, MD USA; 7 Laboratory of Functional and Molecular Imaging, National Institute of Neurological Disorders and Stroke, National Institutes of Health, Bethesda, MD USA

**Keywords:** Multiple sclerosis, Demyelinating diseases

## Abstract

**Background:**

The length, shape, and size of the spinal cord (SC) present unique challenges for MRI, including the need for high resolution to distinguish anatomical and pathological features along its span. Postmortem MRI offers an opportunity to map SC tissue abnormalities and investigate their histological correlates.

**Methods:**

We developed a pipeline combining postmortem microscopic resolution MRI (MR microscopy; MRM) of whole formalin-fixed SC specimens with targeted histopathological analysis. A gadolinium-based tissue preparation protocol was optimized using SC tissue from common marmosets with experimental autoimmune encephalomyelitis. A custom tissue holder and container were designed to enable postmortem MRI of the entire human SC. Human SC samples from individuals with multiple sclerosis, amyotrophic lateral sclerosis, and intracranial hemorrhage were scanned at 75 μm isotropic resolution on a 9.4 T Bruker system after gadolinium preparation.

**Results:**

MRM after gadolinium-based tissue preparation yields images with high signal- and contrast-to-noise ratio while minimizing acquisition times. MRI demonstrates fine anatomical detail and pathological features, including demyelination and neurodegeneration throughout the SC. A complementary custom-made cutting rack enables targeted histological sectioning of MRI-identified regions. This approach provides precise spatial correspondence between imaging and histological findings, demonstrating strong agreement across modalities.

**Conclusions:**

In summary, this pipeline facilitates comprehensive SC assessment by integrating MRM with histology. It enables accurate localization of both subtle and widespread SC pathology and enhances interpretation of MRI signals in the context of neurodegenerative and inflammatory diseases.

## Plain Language Summary

The human spinal cord is generally understudied due to technical difficulties. We provide a method for detailed postmortem imaging of the entire human spinal cord that guides tissue dissection and further microscopic analysis. We optimized this method on spinal cords from marmosets modelling multiple sclerosis and human specimens of different neurological diseases. Using this approach, we acquired detailed images of spinal cord anatomy and abnormalities and were able to precisely extract selected parts for microscopic analysis, with excellent correspondence between postmortem imaging and microscopy. Our method can be used to better understand human spinal cord disorders and abnormalities detectable by imaging and microscopy.

## Abstract (web summary)

Tsagkas, Donadieu et al. provide a pipeline combining ultra-high-resolution postmortem MRI of the entire human spinal cord with MRI-guided histology. The method produces high-contrast images that reveal fine anatomy and disease changes, with strong spatial agreement between MRI findings and tissue pathology.

## Introduction

The spinal cord (SC) can be affected by various neurological disorders with diverse etiologies, including genetic, inflammatory, demyelinating, degenerative, and infectious. SC injury often leads to severe motor, sensory, and autonomic dysfunction^1,2^. Magnetic resonance imaging (MRI) is the primary in vivo tool for the evaluation of SC tissue injury both in research settings and clinical routine. However, the SC’s length, shape, size, physiological movement, and anatomical location pose unique challenges for in vivo MRI^3–5^. As a result, technical constraints have significantly limited our ability to study SC disorders and uncover the underlying pathological mechanisms.

Postmortem MRI of the brain has been used to improve the efficiency of histopathological examinations^6–8^, and has a higher sensitivity to detect pathological changes than visual inspection. Furthermore, postmortem MRI can be performed on the whole organ, providing detailed structural and some quantitative information, as well as the ability to pinpoint regions of interest for histopathology^9–14^. Such experiments have also provided important insights into the relationships between tissue microstructure and the biophysical sources underlying brain MRI contrast^15–18^. Hence, postmortem MRI of the SC could play an equally important role in improving the understanding of neurological diseases.

However, postmortem MRI of the SC is more challenging than the brain, primarily because of its thin but long cylindrical shape with a maximal transverse diameter of 13 mm and a length of approximately 45 cm^19–23^. Prior postmortem MRI studies of SC pathology have been largely confined to short, excised segments, usually between 2 and 6 cm^10–14^. However, imaging small SC segments reduces the interpretability of MRI findings, especially compared with in vivo clinical MRI, and restricts comprehensive analysis. Despite the previous development of robust methodological strategies for combined MRI and histological analysis for brain tissue^6,8,24–30^, there is currently a lack of similar pipelines that can be applied to whole SC specimens.

Due to the SC’s small cross-sectional size, high resolution is required over its entire length for visualization of its fine anatomical structures and tissue abnormalities. MR microscopy has been defined as MRI with resolution in the micron scale and has long been used to study fine anatomical details, not only in postmortem tissue samples but also in vivo in small animals^31–33^. Breakthroughs in gradient design, boosting both maximum strength and slew rates, have allowed for sub-100 μm isotropic resolution in postmortem MRI despite long acquisition times and B_0_ inhomogeneities along the field-of-view (FOV).

In this report, we introduce an innovative pipeline that combines postmortem whole-SC MR microscopy and targeted histopathology. This was achieved through the development of: (i) a human whole-SC cradle fitting the shape of the MR-coil, (ii) a protocol improving image signal- and contrast-to-noise ratios (SNR and CNR) based on immersion in gadolinium, an MRI contrast agent, and (iii) a custom-made cutting rack to dissect SC regions of interest based on postmortem MRI. This method enables detailed visualization of SC anatomy and pathology, while MRI-guided histopathology allows for precise targeting of regions of interest, achieving excellent spatial correspondence between imaging and histological findings.

## Methods

### Optimizing SNR for MR microscopy on marmoset SC

**Fig. 1:**
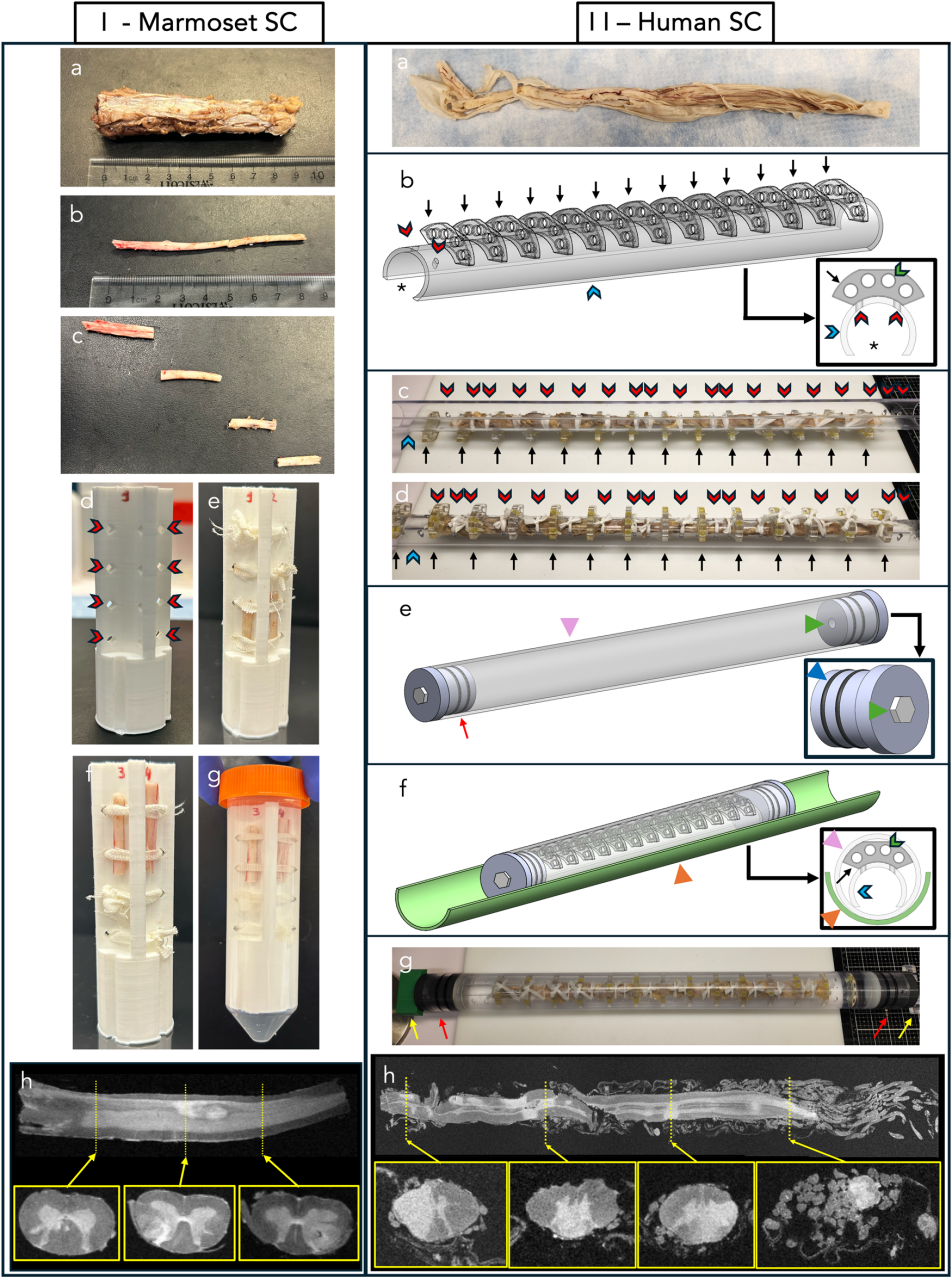
Schematic diagrams of SC imaging cradles and setup for whole marmoset and human SC tissue T2*w MR microscopy. **Ia** After perfusion, the whole spine was extracted en bloc. **Ib** The SC was then extracted by laminectomy from the upper cervical vertebrae to the lower lumbosacral region and **Ic** cut into 4 pieces. **Id** A 3D-printed SC holder was designed with four slots to insert and align all cord pieces, as well as circular openings (red arrowheads) along the lateral aspects of each slot. **Ie**, **f** Each SC piece was secured on the holder using gauze inserted through the cradle’s circular openings. **Ig** The holder was then inserted into a 50 ml plastic Corning centrifuge tube, which was filled with Fomblin. **Ih** MR microscopy of a representative (EAE3) whole marmoset SC depicting relative positions of various demyelinated SC lesions as well as anatomical features (top panel) and reconstructed axial views at various representative levels indicated by yellow lines (bottom row). **IIa** Whole human SC tissue after extraction at autopsy. (IIb-d) Inner tube or tissue holder (blue arrow heads). The tissue holder was a hollow (marked by * in **IIb**), horizontally oriented cylindrical segment. A schematic of the inner tube’s cross-section is shown in the inset image (**IIb**). Wings (black arrows) were placed 4 cm apart along the tissue holder’s length with holes integrated into their design to allow placement of up to 4 MRI fiducial markers (green arrowhead). In addition, the tissue holder has holes (red arrowheads) used to secure the SC in place with gauze strips. **IIe** Outer tube or container (purple arrowhead), with end caps on either side (red arrows, inset image in **IIe**) sealed by O-rings (blue arrowheads) with a sealable hole (green arrowhead) to allow air to egress during setup. **IIf** Schematic of the assembly with the inner tube (blue arrowhead in inset) sealed inside the outer tube (purple arrowhead in inset) and placed on a green MRI-cradle (orange arrowhead). A 3D-printed “guide” was also fitted at each end of the MRI cradle (yellow arrows in IIg) to avoid rotation throughout the course of the scan. **IIh** T2*w MR microscopy of a representative (MS2) whole human SC depicting the relative positions of various demyelinated SC lesions as well as anatomical features (top panel) and reconstructed axial views at various representative levels indicated by yellow lines (bottom row).

To determine the optimal tissue preparation for postmortem MRI, SC were extracted from three adult common marmosets (*Callithrix jacchus*, 2 females, aged 4.7, 3.0, and 3.4 years, detailed demographics in Table 1) with experimental autoimmune encephalomyelitis (EAE) undergoing autopsy in a separate study under a clinical research protocol (protocol number: 1308) approved by the National Institutes of Health Intramural Institutional Review Board, in accordance with the standards of the American Association for Accreditation of Laboratory Animal Care and the National Institute of Neurological Disorders and Stroke (NINDS) Animal Care and Use Committee (Supplementary Methods, Marmoset experimental autoimmune encephalomyelitis). Transcardial perfusion was performed with phosphate-buffered saline under 5% isoflurane anesthesia. In each animal, the spine was extracted en bloc, then fixed by immersion in 10% neutral buffered formalin for 1 week and finally stored in 1% neutral buffered formalin until tissue blocking and histology (Fig. 1–Ia). The SC was then extracted by laminectomy from the upper cervical vertebrae to the lower lumbosacral region and cut into four pieces before MRI acquisition (Fig. 1–Ib-c). Fixation of the spine before SC extraction was preferred over direct extraction at autopsy, as fixation-induced tissue stiffening facilitated laminectomy.

**Table 1:** Demographic and clinical information of all EAE marmosets

Animals	Sex	Age at death (years)	hMOG Dose (μg)	Experiment duration (weeks)	EAE score at terminal^53^
Total cohort, *n* = 3	2 F/1 M	3.7 ± 0.9^a^	50 ± 45.9^a^	7.5 ± 2.0^a^	16 ± 2.3
EAE1	M	4.7	100	5.4	15
EAE2	F	3.0	10	7.9	19
EAE3	F	3.4	40	9.3	15

^a^Mean ± standard deviation.

*EAE* experimental autoimmune encephalomyelitis, *hMOG* human myelin oligodendrocyte glycoprotein, *F* female, *M* male, *n* number.

All marmoset whole-SC tissue underwent a gadolinium-based preparation, similar to previous studies^9,34,35^. This preparation shortens the tissue’s T_1_ relaxation time, thus enabling the use of shorter repetition times (TR) and increasing the SNR partly by allowing more acquisitions in a given total scan time. To optimize SNR while preserving anatomical details, three sets of experiments were performed on marmoset SCs. First, the four SC pieces from the first marmoset (EAE1) were immersed for 2, 4, 6, or 8 days in a 4 mM solution of gadolinium (Gadavist, Bayer) diluted in phosphate-buffered saline (Gad). Similarly, SC pieces from a second marmoset (EAE2) were immersed for 8 days in 0-, 1-, 2-, or 3-mM Gad-solution. Finally, SC pieces from a third marmoset (EAE3) were immersed for 8 days in 1.2-, 1.6-, 2.0-, or 2.4-mM Gad solution. Marmoset SC pieces were then positioned in a custom-made SC holder (Fig. 1–Id-f), similar to a previous study^36^. This holder was designed (Netfabb Professional 5.0 software, Autodesk Inc., San Rafael, CA, USA) with four slots to insert and align all SC pieces, as well as circular openings along the lateral aspects of each slot to secure the SC pieces using gauze strips (Fig. 1–Id). The holder was then inserted into a 50 ml plastic Corning centrifuge tube (Millipore Sigma, CLS430291), which was then filled with Fomblin 06/6 (Specialty Fluid Company, Castaic, CA, USA), a perfluoropolyether that lacks MR signal but has tissue-matched susceptibility, thereby reducing image artifacts (Fig. 1–Ig).

T2*-weighted (T2*w) postmortem SC MRI of EAE1 and EAE2 was performed on a 7-T Bruker (Biospec 7 T/ 30 cm) scanner using a 30-mm inner diameter quadrature coil (Millipede coil, ExtendMR LLC). Postmortem SC MRI of EAE3 was performed on a 9.4-T Bruker (Biospec 9.4 T/30 cm) scanner using an 86-mm transmit-receive volume coil (Fig. 1–Ih). Imaging protocols are detailed in Table 2, with MR microscopy sequences annotated with an asterisk. SNR was calculated as the ratio of the average signal from various tissue compartments (i.e., normal-appearing and lesional gray and white matter; NAGM, NAWM) to the standard deviation of noise in the background. CNR was calculated as the difference between the SNR from various tissue compartments.

**Table 2:** MRI acquisition parameters

Sequence	TR (ms)	TE (ms)	FA (°)	Scan time (min per average)	Acq. Matrix (RL x AP X SI)	Resolution (μm)	Averages
Marmoset SC EAE: postmortem 7 T MRI							
T2*w 3D GRE*	50	6	14	147	420 ×400×860	60 ×60×60	5-7
Marmoset EAE SC: postmortem 9.4 T MRI							
T2*w 3D GRE*	40	6	15°	136	400 ×400×512	75 ×75×100	5
Human SC: pre-gadolinium postmortem 9.4 T MRI							
T2*w 3D GRE	40	20	15°	80	300 ×300×400	100 ×100×200	1
T2*w 3D multi-echo GRE*	50	4, 9, 14, 19, 24, 29	17°	53.1	266 ×266×240	75 ×75×100	~20
Human SC: post-gadolinium postmortem 9.4 T MRI							
T2*w 3D GRE	40	9	30°	11.5	125 ×125×138	400×400×400	1
T2*w 3D GRE*	45	9	44°	45.9	306 ×200×730	75×75×75	12-21

Averages were adjusted based on total scanner time available and length of the SC.

*EAE* experimental autoimmune encephalomyelitis, *GRE* gradient echo, *Acq.* acquisition, *AP* anterior-posterior axis, *RL* right-left axis, *SI* superior-inferior axis, T2*w T2*-weighted. *Min* minimum. *denotes MR microscopy sequences. *AP* anterior-posterior axis, *RL* right-left axis, *SI* superior-inferior axis.

### MRI tissue holder and cradle for human SC

A human SC tissue holder and container were designed to enable MRI of the whole cord. The tissue holder (inner tube, polycarbonate) was a hollow, horizontally oriented cylindrical segment measuring 63 cm in length, with an inner diameter of 3.2 cm and a wall thickness of 0.3 cm. Pairs of holes were drilled at regular intervals along its length to secure the SC in place using gauze strips (Fig. 1–IIa–d). 3D printed wings of 0.6 cm thickness were mounted on the outer surface of the inner tube at 4 cm intervals to stabilize the holder within the container. Four holes were also integrated into the design of each wing (Fig. 1–IIa–d), allowing placement of MRI fiducial markers (Beekley PinPoint #128 markers). The tissue holder was designed to be placed in a hollow cylinder (outer tube or container, polycarbonate, Fig. 1IIe–g) filled with Fomblin and measured 66 cm in length, with an inner diameter of 5.1 cm and a wall thickness of 0.3 cm. End caps for the container were designed with O-rings to make it air-tight, and with a sealable threaded hole in the center to allow for egress of air during setup (Fig. 1–IIe–g). This hole also allowed for topping up Fomblin levels after closing the container, as well as for the application of gentle suctioning to remove air bubbles stuck to the tissue surface. After setup, the hole was closed using a thumb screw. A guide fitted to the external surface of the container prevented rotation during advancement in the scanner (Fig. 1–IIg).

### MR microscopy of human SC

SCs from three multiple sclerosis (MS), one amyotrophic lateral sclerosis (ALS), and one intracranial hemorrhage (ICH) were obtained at autopsy with consent from the next of kin (detailed demographics in Table 3). All SCs were extracted, immersion-fixed in 10% neutral buffered formalin for 1–2 weeks, and subsequently stored in 1% neutral buffered formalin until imaging, tissue blocking, and histology. For the three MS cases (MS1-3), demographic, clinical information, and in vivo MRI scans were obtained after informed consent under a clinical research protocol (protocol number: 89-N-0045; ClinicalTrials.gov number: NCT00001248:1992-07-23) approved by the National Institutes of Health Intramural Institutional Review Board and were compared with the postmortem imaging. For cases 4 (amyotrophic lateral sclerosis, ALS) and 5 (intracranial hemorrhage, ICH), demographic and clinical information were obtained through the National Disease Research Interchange (https://ndriresource.org/, protocol ID RRED1 01 002A) under a tissue recovery protocol customized for the purposes of this project. No additional IRB approval was obtained since only shipping approvals are needed for postmortem samples. T2*w postmortem MRI of the human SC was performed on a 9.4-T Bruker (Biospec 9.4 T/30 cm) scanner using an 86-mm transmit-receive volume coil (Fig. 1–IIh). Optimized imaging protocols are detailed in Table 2, and the sequence optimization process is described in the Supplementary Methods (MRI sequence optimization) and Supplementary Fig. 1. To achieve full cord coverage, the SC was scanned in multiple segments (12 repetitions minimum per segment; up to 9 segments required to scan the entire SC), each with a 5.5-cm superior-inferior FOV and a 0.5-cm overlap between subsequent segments to avoid unscanned SC areas and facilitate consecutive image stitching. The container was advanced in the scanner using a ruler after MRI acquisition of each SC segment (roughly every 9.2 h), and the scan was repeated until the entire SC was scanned. Each SC was scanned before and after incubation in 1.6 mM for 8 days in Gad solution. Fomblin was replaced regularly.

**Table 3:** Demographic and clinical information of all human autopsy cases

Case	Sex	Age at death (years)	Diagnosis	PMI (hours)	Cause of death	SC segments extracted	SC rostrocaudal length (cm)	Interval between last in vivo SC MRI and death (years)
Total cohort, *n* = 5	2 F/3 M	63.8 ± 11.5^a^	3 PMS, 1 ALS, 1 ICH				30.0 ± 13.6^a^	4.6 ± 4.2^a^
MS1	F	75–80	PMS	<24	Ischemic stroke	C1 — Co1	42.1	3.4
MS2	F	60–65	PMS	8	Acute polymicrobial bronchopneumonia	T2 — Co1	28.9	1.2
MS3	M	70–75	PMS	<20	Unknown	C3 — Co1	42.6	9.3
ALS1	M	45–50	ALS	<24	Anoxia	L1 — Co1	9.5	—
CVD1	M	60–65	ICH	37.5	ICH	T2 — Co1	27.0	—

^a^ Mean ± standard deviation.

*MS* multiple sclerosis, *ALS* amyotrophic lateral sclerosis, *CVD* cerebrovascular disease, *ICH* intracranial hemorrhage, *F* female, *M* male, *n* number, *PMI* postmortem interval, *PMS* progressive MS, *C* cervical, *T* thoracic, *L* lumbar, *Co* coccygeal.

**Fig. 2:**
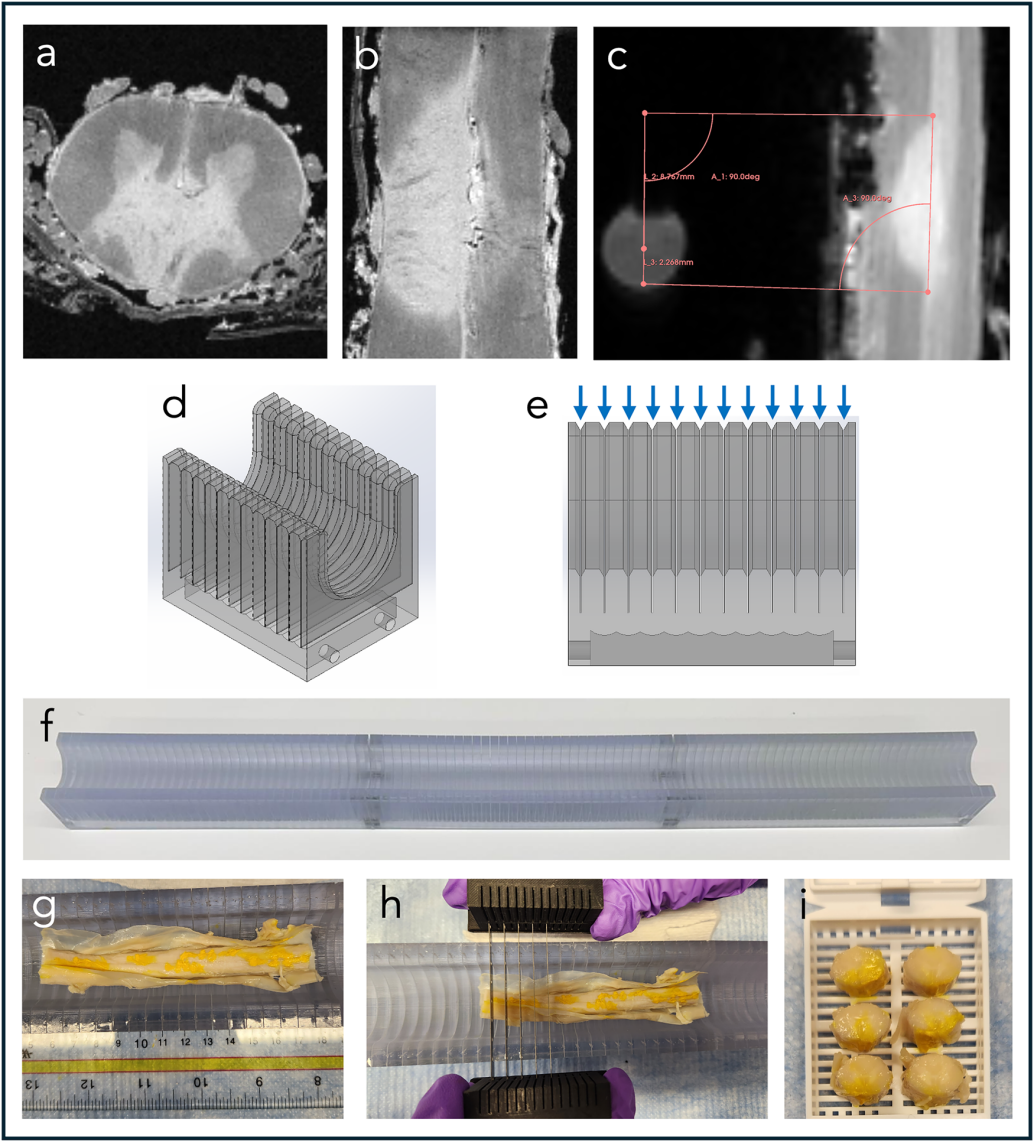
MRI-guided histology. T2*w MR microscopy of a representative MS SC lesion on the axial (**a**) and sagittal (**b**) views. **c** The distance between the rostrocaudal borders of each region of interest from the nearest fiducial marker was measured on T2*w postmortem MRI of the same tissue with lower resolution. **d**–**f** The SC cutting cradle mimics the MRI tissue holder except for 0.3mm-wide gaps for blades (blue arrows) placed 6 mm apart. **g**–**i** The borders of all regions of interest were drawn on the specimen using yellow tissue marking dye (visible on SC tissue in **g** and **h**). Subsequently, the SC was moved to the cutting cradle (**g**), where slices were cut consecutively from the rostral to the caudal SC end using a 3D-printed blade holder (black pieces in **h**). **i** Up to 6 tissue slabs were transferred to a cassette for paraffin embedding and labeled immediately.

T_2_*w MRI repetitions from the same scanned SC FOV were averaged using software adapted for MRI image processing: Medical Image Processing, Analysis and Visualization (https://mipav.cit.nih.gov/). The rostrocaudal borders of regions of interest (ROI) (e.g., MS lesions) were identified using 3D Slicer (version 5.2.2) by measuring their distance from the nearest fiducial markers on the 400 μm isotropic resolution T_2_*w MRI sequence (fiducial markers included in FOV, Fig. 2a–c). Then, MR microscopy images of SC segments were stitched together using the fiducial markers as a guide (Fig. 1–IIh).

### MRI-guided histopathology

The distances from each ROI’s rostrocaudal borders to the nearest cradle fiducial markers were measured on MRI and then used to accurately mark each ROI on the SC tissue with a fine brush and yellow marking dye (Mopec, BG109-60), without removing the SC from the tissue holder. Subsequently, the SC was moved to a custom-made cutting cradle designed for precise sectioning of the SC. The cutting-cradle was made of three identical segments screwed together, with a hollow cylindrical upper surface (Fig. 2d–f). Each segment was 21.4 cm long and had 34 0.3mm-wide blade slits spaced at 6-mm intervals (Fig. 2f). 6mm-thick tissue slabs were then cut consecutively from the rostral to the caudal SC end (Fig. 2g–h). Up to 6 blocks at a time were loaded into a cassette and embedded in paraffin (Fig. 2i). Correspondence between SC slabs and ROI was documented throughout this procedure.

Five-micron-thick sections were obtained from paraffin blocks using a microtome (Leica RM2235, Leica Biosystems) and mounted on glass slides. Sections were chemically stained with hematoxylin and eosin (H&E) and Luxol fast blue-periodic acid Schiff (LFB-PAS), as well as using antibodies against myelin proteolipid protein (PLP, Bio-Rad MCA839G), and were then visually compared with their MRI counterpart. For PLP staining, sections were deparaffinized and rehydrated before being rinsed in triphosphate-buffered saline (TBS) for 10 min, processed for 15 min at 95 °C for antigen retrieval (heat-induced epitope retrieval), and then treated with 3% endogenous peroxidase blocking for 10 min. The sections were blocked with a protein blocking solution (Dako, X090930-2) for 20 min and incubated with primary antibodies for 60 min at room temperature. The sections were then rinsed with TBS+Tween 20 and incubated with anti-mouse horseradish peroxidase-conjugated secondary antibodies (Leica Biosystems, PV6114) for 30 min. 3,3’-DiaminoBenzidine horseradish peroxidase was used for PLP development (2 to 4 min). Stained sections were digitally scanned using a digital slide scanner (NanoZoomer® S20 Slide scanner system, Hamamatsu) and processed using the NDP.view2 software (U12388-01, Hamamatsu).

## Results

### Optimal gadolinium exposure

**Fig. 3:**
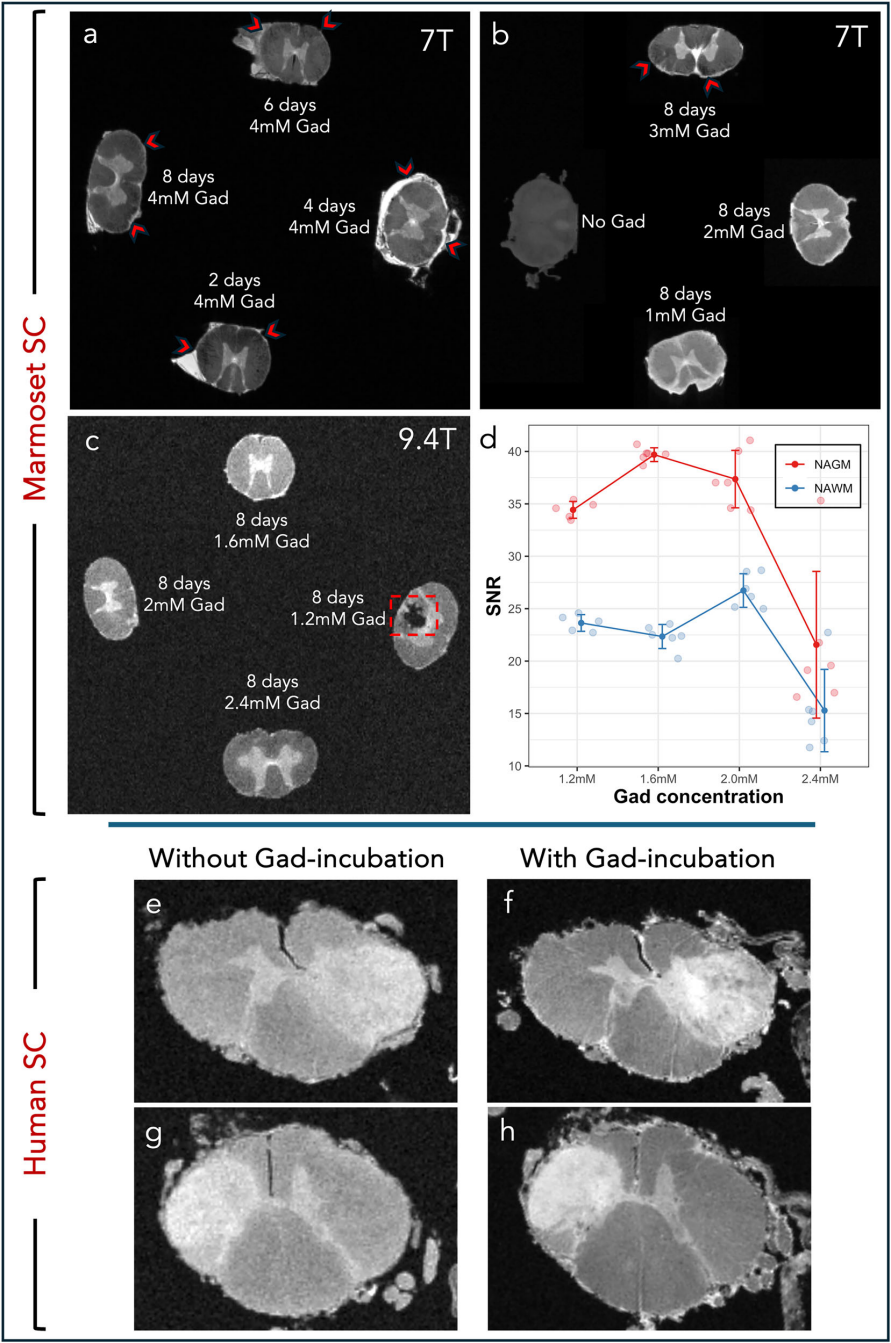
Optimization of gadolinium exposure for marmoset EAE SC tissue. **a** The four SC pieces of one EAE marmoset (EAE1) were immersed in a gadolinium solution (Gad) with a concentration of 4 mM for 2, 4, 6, and 8 days and then scanned at 7 T. **b** Next, the four SC pieces of a second EAE marmoset (EAE2) were immersed in a gadolinium solution with concentrations of 0, 1, 2, and 3 mM for 8 days and then scanned at 7 T. Gadolinium concentrations ≥3 mM led to signal inhomogeneities in the marmoset EAE SC (**a**, **b**; red arrowheads). **c** The four SC pieces of a third EAE marmoset (EAE3) were immersed in a gadolinium solution with concentrations of 1.2, 1.6, 2, and 2.4 mM for 8 days and then scanned at 9.4 T. The red dashed square shows a SC hemorrhagic lesion detected in EAE3. **d** Mean normal-appearing gray and white matter (NAGM; NAWM) signal-to-noise ratio (SNR) ± standard deviation of SC tissue scanned at 9.4 T and incubated at various Gad concentrations (number of samples for each tissue type and Gad concentration = 6). Comparison of postmortem T2*w MRI of human SC before (**e**–**g**) and after (**f**–**h**) incubation in 1.6 mM gadolinium in phosphate-buffered saline (Gad-incubation) for 8 days, showing improved visualization of structural and pathological features and enhanced contrast between SC compartments. **e**, **g** MRI without Gad-incubation was acquired using a 3D T_2_*-weighted multi-echo gradient echo sequence with an in-plane resolution of 75 × 75 μm^2^, a slice thickness of 100 μm, and 48 averages. **f**, **h** MRI after Gad-incubation was acquired using a 3D T_2_*-weighted gradient single-echo sequence with an isotropic resolution of 75 μm and 20 signal averages.

In order to improve SNR and shorten acquisition times of T2*w MR microscopy scans, we explored the use of tissue pre-incubation in a Gad solution using marmoset EAE SC. First, to determine the homogeneity of Gad absorption, a relatively high concentration (4 mM) was used with incubation periods varying from 2 to 8 days (Fig. 3a, and Table 4). In T2*w MR microscopy images acquired at 7 T, signal inhomogeneities were observed within the SC WM for incubation periods <6 days. A more homogenous signal across the SC was observed for incubation periods of 6 and 8 days (Fig. 3a). Therefore, incubation periods of 8 days were chosen for all subsequent experiments.

**Table 4:** SNR and CNR of marmoset SC tissue at 7 T after incubation with different gadolinium-solutions for variable incubation periods

Tissue/Gad preparation	SNR NAWM ^a^	SNR NAGM ^a^	CNR ^a^ NAWM-NAGM
Marmoset SC – 4 mM Gd for 2 days	44.6 ± 6.0	83.8 ± 18.5	39.2 ± 18.0
Marmoset SC – 4 mM Gd for 4	79.9 ± 8.5	128.8 ± 2.6	48.9 ± 8.1
Marmoset SC – 4 mM Gd for 6 days	60.4 ± 7.0	117.5 ± 18.3	57.1 ± 18.1
Marmoset SC – 4 mM Gd for 8 days	61.9 ± 8.7	93.4 ± 14.5	31.5 ± 15.7
Marmoset SC – no Gd	48.3 ± 1.6	54.5 ± 1.9	6.1 ± 2.2
Marmoset SC – 1 mM Gd for 8 days	69.0 ± 4.9	93.9 ± 6.3	24.9 ± 7.4
Marmoset SC – 2 mM Gd for 8 days	45.0 ± 5.0	79.7 ± 5.2	34.7 ± 6.6
Marmoset SC – 3 mM Gd for 8 days	43.9 ± 6.0	64.6 ± 10.2	21.6 ± 10.8

^a^ Mean ± standard deviation.

*Gad* gadolinium, *SNR* signal-to-noise-ratio, *CNR* contrast-to-noise-ratio, *SC* spinal cord, *NAWM* normal-appearing white matter, *NAGM* normal-appearing Gray matter.

Next, to determine SNR increase related to gadolinium, marmoset EAE SC tissue was incubated in Gad concentrations of 0 to 3 mM and imaged at 7 T (Fig. 3b). While there was a 43% SNR boost between 0- and 1-mM Gad experiments, SNR decreased due to shortening of T_2_ relaxation time at Gad concentrations ≥2 mM (Fig. 3b, and Table 4). Signal inhomogeneities were also observed at 3 mM (Fig. 3b). Finally, we titrated the Gad concentration between 1.2 and 2.4 mM, keeping the incubation period at 8 days, but performed scanning at 9.4 T to further increase SNR. (Fig. 3c). Results revealed an SNR peak at 1.6-mM Gad for NAGM, and at 2 mM for NAWM, while CNR between NAGM and NAWM was ≥60% higher at 1.6-mM Gad compared to all other concentrations (Fig. 3c, d, and Table 5). Given this, we chose an incubation time of 8 days in 1.6-mM Gad solution for all subsequent human SC scans.

**Table 5:** SNR and CNR of marmoset SC tissue at 9.4 T after incubation with different gadolinium-solutions

Tissue/Gad preparation	SNR NAWM ^a^	SNR NAGM ^a^	CNR ^a^ NAWM-NAGM
Marmoset SC – 1.2 mM Gd	23.6 ± 0.8	34.4 ± 0.8	10.8 ± 1.0
Marmoset SC – 1.6 mM Gd	22.3 ± 1.1	39.7 ± 0.7	17.3 ± 1.2
Marmoset SC – 2.0 mM Gd	26.7 ± 1.6	37.4 ± 2.7	10.6 ± 2.9
Marmoset SC – 2.4 mM Gd	15.3 ± 3.9	21.6 ± 7.0	7.4 ± 6.3
*p*-value vs 1.6 mM Gd ^b^	1.2 mM Gd: 0.986 2.0 mm Gd: 0.127 2.4 mm Gd: 0.0027	1.2 mM Gd: 0.056 2.0 mm Gd: 0.762 2.4 mm Gd: 3.1×10⁻^11^	1.2 mM Gd: 2.3×10⁻^9^ 2.0 mm Gd: 2.1×10⁻^11^ 2.4 mm Gd: 2.9×10⁻^21^

^a^Mean ± standard deviation.

^b^Mean analysis of covariance with correction using the Tukey HSD (Honestly Significant Difference) method.

*Gad* gadolinium (immersion for 8 days), *SNR* signal-to-noise-ratio, *CNR* contrast-to-noise-ratio, *SC* spinal cord, *NAWM* normal-appearing white matter, *NAGM* normal-appearing Gray matter.

### MR microscopy displays human SC anatomy and pathology in great detail

After an 8-day incubation in 1.6-mM gadolinium concentration, T2*w MR microscopy of whole human SC tissue was attainable at 75-μm isotropic resolution with high SNR in all SC compartments. Comparison of T2*w MR microscopy images before (Fig. 3e, g) and after incubation of SC tissue in Gad for 8 days (Fig. 3f, h) showed improved visualization of SC anatomy and pathology in greater detail. We observed a 2.3- to 14.6-fold CNR increase in pairwise comparisons between the lesional and normal-appearing GM and WM after Gad incubation respectively (Table 6).

**Table 6:** SNR and CNR of MS SC tissue at 9.4 T with and without gadolinium-preparation

Tissue	Without Gad-preparation ^a, b^	With Gad-preparation ^a, c^	*p*-value ^d^
SNR NAWM	31.4 ± 0.2	21.2 ± 1.1	1.4×10⁻^18^
SNR NAGM	35.2 ± 0.3	33.4 ± 0.6	0.010
SNR lesional WM	36.3 ± 0.2	32.7 ± 2.2	1.8×10⁻^6^
SNR lesional GM	37.1 ± 0.3	44.4 ± 1.0	3.5×10⁻^13^
CNR NAWM-NAGM	3.7 ± 0.3	12.2 ± 1.2	2.4×10⁻^75^
CNR NAWM-lesional WM	4.9 ± 0.2	11.5 ± 2.3	3.0×10⁻^60^
CNR NAGM-lesional GM	1.9 ± 0.4	11.0 ± 1.1	3.7×10⁻^159^
CNR lesional WM-lesional GM	0.8 ± 0.3	11.7 ± 2.2	3.1×10⁻^96^

*Gad* gadolinium (1.6 mM for 8 days), *SNR* signal-to-noise-ratio, *CNR* contrast-to-noise-ratio, *SC* spinal cord.

^a^ Mean ± standard deviation.

^b^ MRI acquired at 75 μm isotropic in-plane resolution, with a 100 μm slice thickness.

^c^ MRI acquired at 75 μm isotropic resolution.

^d^ Mean analysis of covariance with correction using the Tukey HSD (Honestly Significant Difference) method.

**Fig. 4:**
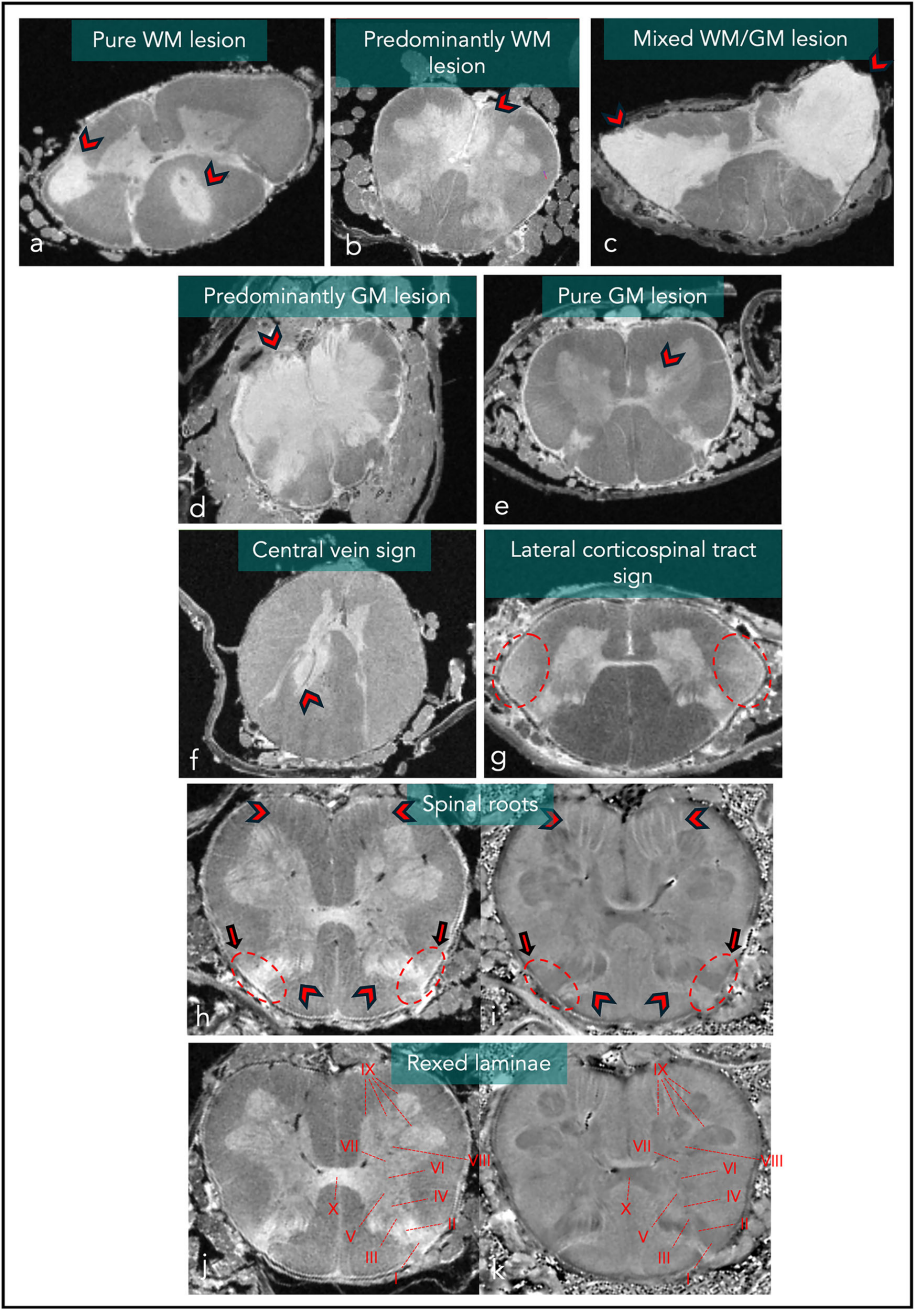
T2*w MR microscopy of human SC enables the visualization of fine anatomical and pathological features. MS SC tissue with demyelinated lesions within different SC compartments, such as pure white matter lesions (**a**), predominantly white matter lesions (**b**), mixed gray and white matter lesions (**c**), predominantly gray matter lesions (**d**), and pure gray matter lesions (**e**). **f** T2*w MR microscopy enabled the clear visualization of the central vein sign in MS SC tissue (red arrowhead). **g** The lateral corticospinal tract sign was detected in the amyotrophic lateral sclerosis SC (red arrowheads). Fine anatomical structures, such as the ventral and dorsal spinal roots (**h**, **i**; red arrowheads), Lissauer’s tract (**h**, **i**; red arrows), and the Rexed laminae (**j**, **k**; annotations), were visible. WM: white matter. GM: gray matter.

Acquisition of T2*w magnitude and phase images allowed for detailed 3D examination of the SC (Fig. 4a–k), enabling the detection of: (I) various demyelinating lesions, including small pure gray matter lesions (Fig. 4e); (II) neurodegenerative pathology, such as the lateral corticospinal tract sign (Fig. 4g); (III) small vascular structures and the central vein sign within MS lesions (Fig. 4f); (IV) ventral and dorsal spinal roots (Fig. 4h, i); and (V) the Rexed laminae (Fig. 4j, k), including distinct ventral motor nuclei^37–39^.

### MRI-guided histopathology

**Fig. 5:**
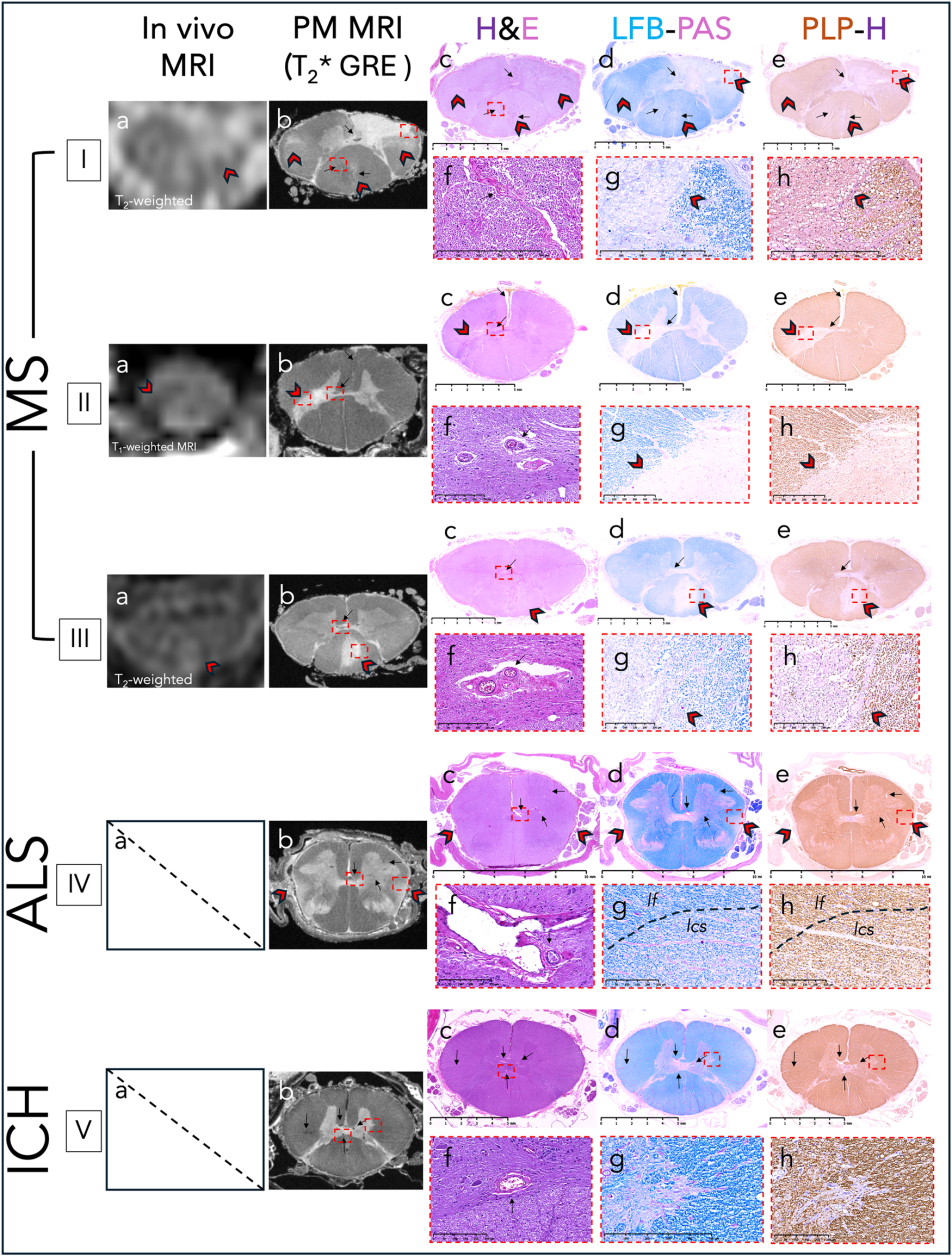
From in vivo MRI to MR microscopy and histopathological analysis. **Ia**-**IIIh** Representative examples of MS SC lesions detected both on T_1_- or T_2_-weighted in vivo MRI (**I**/**II**/**IIIa**), and T2*w postmortem MRI (**I**/**II**/**IIIb**). **IVa**–**Vf** Representative examples of ALS and ICH SC tissue imaged on T2*w postmortem MRI, while in vivo MRI was not available (**IV**/**Va**). Histological sections of whole transverse SC sections (**I**/**II**/**III**/**IV**/**Vc**–**e**) and magnified regions shown in red dashed rectangles (**I**/**II**/**III**/**IV**/**Vf**–**h**) stained with hematoxylin and eosin (**I**/**II**/**III**/**IV**/**Vc**, **f**), Luxol fast blue combined with periodic acid-Schiff (**I**/**II**/**III**/**IV**/**Vd**, **g**), and myelin proteolipid protein (PLP) immunohistochemistry (**I**/**II**/**III**/**IV**/**Ve**, **h**) corresponding to respective postmortem MRI (**I**/**II**/**III**/**IV**/**Vb**). Red arrowheads indicate the correspondence between features of demyelinating and neurodegenerative pathology on T2*w MRI and histology. Numerous demyelinated lesions were found in MS SC tissue (**I**/**II**/**IIIg**, **h**), while ALS SC exhibited bilateral degeneration of the lateral cortico-spinal tract (**IVg**, **h**). Black arrows indicate the correspondence between vessels seen both on T2*w MRI and histology. ALS amyotrophic lateral sclerosis, MS multiple sclerosis, ICH intracranial hemorrhage, PM postmortem, H&E hematoxylin and eosin, LFB-PAS Luxol fast blue combined with periodic acid-Schiff, PLP myelin proteolipid protein, LCS lateral corticospinal tract. lf: lateral funiculus. Scale bars in **I**/**II**/**III**/**Vc**–**e**: 5 mm; in **IVc**–**e**: 10 mm; in **If**–**h** & **Vg**: 500 μm; in **II**/**III**/**IV**/**f**–**h** & **Vf**, **h**: 250 μm.

T2*w postmortem MR microscopy images were used to systematically document imaging features and precisely guide pathological sectioning of ROIs, thereby increasing the efficiency of histological analysis efficiency (Fig. 2). The overall SC anatomy, as well as the morphology and location of demyelinated MS lesions and neurodegenerative changes, were consistently and accurately reflected in both T2*w MRI and histology (Fig. 5, and Supplementary Fig. 2). Of note, even small vascular structures within the SC tissue were seen in both MR microscopy and histological images (Fig. 5I-Vc, f), demonstrating the precision of the MRI-histological matching.

## Discussion

In this work, we present a pipeline combining T2*w MR microscopy of whole formalin-fixed SC specimens with targeted histopathological analysis. This approach enabled precise, image-guided localization of regions of interest, substantially enhancing the accuracy and efficiency of histological analysis in human SC tissue.

T2*w postmortem MRI of whole human SC tissue was attainable at microscopic resolution (75-µm isotropic voxels) using a single tissue preparation step. Incubation of SC in 1.6 mM Gad for 8 days significantly increased SNR and improved visualization of fine anatomical and pathological details. This imaging approach facilitated robust MRI-histopathology correspondence, enabling the detection of subtle and spatially confined SC pathological features. Precise alignment between histological sections and T2*w MRI data can be achieved rapidly owing to prior documentation of ROI-to-tissue slab correspondence, obviating the need for additional image reformatting or resampling. ROIs, including demyelinated lesions identified in vivo and T2*w postmortem MRI, were validated by gold-standard histological staining, demonstrating the translational potential of this approach to link imaging features with microscopic pathology.

The main challenge for high-resolution MRI of the SC is its shape (long, thin cylinder). To gain insights into SC pathology, previous studies using postmortem MRI focused on relatively small SC pieces (2–6 cm) to map local SC abnormalities^10–14^. However, imaging of whole SC tissue — analogous to whole brain sampling — offers additional benefits, such as preservation of SC anatomy, global overview of SC pathology for each case, and reduction of MRI artifacts commonly occurring at the tissue surface^40–42^. Imaging the whole SC in its native state also avoids the need for blind cutting of the SC into segments, which can introduce distortions, damage tissue edges, and complicate direct comparisons with in vivo MRI.

Due to the SC’s long and thin morphology as well as scanner constraints (RF coil profile, gradient linearity, image size, and inhomogeneous field areas), imaging was performed in overlapping segments of 55 mm. Our home-built SC holder enabled precise positioning in the magnet, and together with fiducial markers — which provided reference when intrinsic landmarks were insufficient — facilitated stitching of MRI across individual SC segments. Future integration of motorized devices such as the patient table used in clinical scanners could further improve the accuracy of this approach.

Our imaging protocol included optimized T2*w 3D-GRE sequences with acquisition times under an hour per average, both for a 55-mm Gad-incubated SC segment (75 µm isotropic) and for a 24-mm non-incubated segment (75 µm isotropic in-plane, 100 µm slice thickness). The protocol was mainly optimized based on the relaxation tissue properties of MS SC lesions and WM. However, postmortem interval, associated diseases, and formalin fixation duration can alter tissue relaxation times,^41,43–45^ and a single protocol might not be ideal for all SC samples, although these factors could be partially mitigated by control of postmortem interval before fixation or by selection of SC specimens within a postmortem interval range, as well as standardizing fixation protocols and minimizing fixation times. Nevertheless, we were able to obtain consistent image quality from both pre- and post-Gad incubation in all our SC specimens.

Several technical limitations remain with this approach of SC imaging. In addition to several factors generally affecting postmortem MR signal,^43–46^ the absence of multimodal MRI contrasts, the high spatial resolution (which is currently unattainable in vivo), and the reliance of our technique on gadolinium-based contrast enhancement hamper the clinical translation of our imaging approach. However, the translational potential of this method is related to its capability to: (i) validate in vivo structural MRI biomarkers (especially in the scenario in which both in vivo and postmortem images are available); (ii) discover previously undetected or under-detected pathology (which may be highly relevant in the spinal cord due to the current limitations of in vivo MRI); and (iii) potentially understand disease mechanisms and the cord’s spatial biology. Furthermore, advanced MRI sequences such as diffusion MRI are feasible in the postmortem setting^12,15,26,46,47^— as demonstrated in previous studies using a gadolinium-based tissue preparation — but are constrained by exceptionally long scan times^9^. For example, diffusion-weighted imaging of an 8 cm spinal cord segment required approximately 37.8 h of acquisition time^9^. Postmortem quantitative SC mapping (T_1_ and T_2_ relaxivity) would also be attainable with a refined imaging protocol, but must be performed before gadolinium immersion to avoid drastic changes in tissue relaxation. While gadolinium preparation shortens the tissue’s T_1_ relaxation time and indirectly reduces acquisition time, it also shortens T_2_ relaxation times, necessitating shorter echo times for optimal signal sampling, which can be challenging at high resolutions^9,34,35^. Moreover, the impact of gadolinium preparation on the detection of certain pathological features in phase MRI is not addressed here, though preliminary data suggest close correspondence between pre- and post-Gad incubation SC MRI (Fig. 3e–h). Finally, in this study, we were able to visually identify the MRI slices corresponding to our histological sections. This was facilitated by the combined use of our custom-made SC tissue holder, container, and cutting rack that enabled the acquisition of both axial MRI slices and histological sections perpendicular to the rostrocaudal SC axis, as well as by the use of fiducial markers during MRI acquisition. However, a more accurate identification of the optimal histology-corresponding MRI slice could be achieved through the addition of non-linear registration to the pipeline, as previously implemented in postmortem brain blocks,^26,48–52^ although such methods have not yet been validated in the SC.

To conclude, we developed a method for T2*w MR microscopy of the entire human SC as an accurate guide for histopathological examinations. This method provides high-resolution single-echo T2*w GRE images that allow for identification of pathological alterations in the human SC, such as demyelinated lesions and neurodegeneration. Those changes can then be extensively and accurately studied using histological techniques. This approach provides a strong link between in vivo imaging and microscopic pathology, with the potential to deepen our understanding of the pathologic substrate of MRI abnormalities and their relationship with disease mechanisms in various neurological disorders affecting the SC.

## Supplementary information

Transparent Peer Review file

Supplementary material

Description of Additional Supplementary files

Supplementary Data File 1.

Supplementary Data File 2.

Supplementary Data File 3.

Supplementary Data File 4.

Supplementary Data File 5.

## Data Availability

Source data for Fig. 3 and Tables 4–6 of this study can be found in Supplementary Data File 1. Source data for Supplementary Fig. 1 can be found in Supplementary Data Files 2–5. The authors declare that all data and materials will be available upon reasonable request. The authors also declare that all other data supporting the findings of this study are available within the paper and its supplementary information files.
