# Supplementary material for: A pipeline to characterize spinal cord pathology in neurological disorders combining magnetic resonance microscopy and histopathology
Source: Commun Med (Lond). 2026 Apr 22;6:370. doi: 10.1038/s43856-026-01577-8 (PMC13324037; doi:10.1038/s43856-026-01577-8)
Supplement: Supplementary file 2 — Supplementary material [file 43856_2026_1577_MOESM2_ESM.pdf]

Supplementary Material

Supplementary Methods

Marmoset experimental autoimmune encephalomyelitis

All animals were induced with experimental autoimmune encephalomyelitis (EAE) using 10 to 100µg of human myelin oligodendrocyte glycoprotein (hMOG) emulsified in incomplete Freund's adjuvant (Difco Adjuvant, 263910, BD) in a 1:1 volumetric ratio. A total of 200µL homogenate was injected subcutaneously at one or two of four dorsal sites around the axillary lymph nodes. All animals were sacrificed following the National Institute of Neurological Disorders and Stroke (NINDS) Animal Care and Use Committee guidelines when either the body weight decreased more than 10%, both legs were paralyzed, or the animal experienced severe hypothermia.

MRI sequence optimization

To optimize our T2*-weighted MRI sequence, we experimented with basic sequence parameters such as the echo time, the repetition time, the flip angle, and the repetitions acquired for each FOV (Supplementary Fig. 1). First, we observed increased SNR and marginally increased CNR using a TE of 6ms compared to 10ms in the marmoset spinal cord. In a similar experiment of multiple sclerosis spinal cord tissue using TE of 8-10ms (lower TE were not feasible at 100 µm isotropic resolution due to MR system limitations), we observed slightly increased SNR for spinal cord compartments between 8ms and 10ms (normal-appearing and lesional WM: $p < 0.01$; normal-appearing and lesional GM: $p < 0.05$). Hence, for MR microscopy at 75 µm isotropic resolution, we selected the lowest feasible TE value of 9ms. Next, using a TE of 9ms, we assessed the effect of TR values between 25-70ms. At 100 µm isotropic resolution, we observed a significantly higher SNR with higher TR values (all $p < 0.001$), but a smaller increase in CNR using a TR of 70ms compared to 50ms (normal-appearing WM/GM to lesional WM/GM: $p < 0.5$; normal-

appearing WM to normal-appearing GM: not significant; lesional WM to lesional GM: not significant). Since longer TR values came at a cost of longer acquisition times, we selected a TR of 45ms for MR microscopy at 75 μm isotropic resolution. To calculate the optimal flip angle, we acquired, before gadolinium-based tissue preparation, magnetization-prepared rapid acquisition gradient echo images (300 μm isotropic resolution, TR=4000ms, TE=1ms, flip angle 8°) at multiple TI: 50ms, 100, 150ms, 200ms, 300ms, 400ms, 500ms, 600ms, 800ms, 1000ms) and generated spinal cord T1 maps. We calculated a T1 value of 123.8 ± 1.3 at the spinal cord gray matter, corresponding to a flip angle ≈ 46° for TR = 45 ms using the Ernst equation, while lesions had variably longer T1 values. To obtain maximum signal from the spinal cord gray matter and lesions, we subsequently experimented with flip angles slightly lower than 46°, and observed slightly higher SNR and CNR at 44° compared to 39° (difference not statistically significant). Finally, we calculated the number of repetitions required in each field-of-view for SNR maximization. Since there was minimal SNR gain after 12 repetitions at 75 μm isotropic resolution, we selected this as the minimum number of repetitions for each field-of-view in our experiments.

Supplementary figures

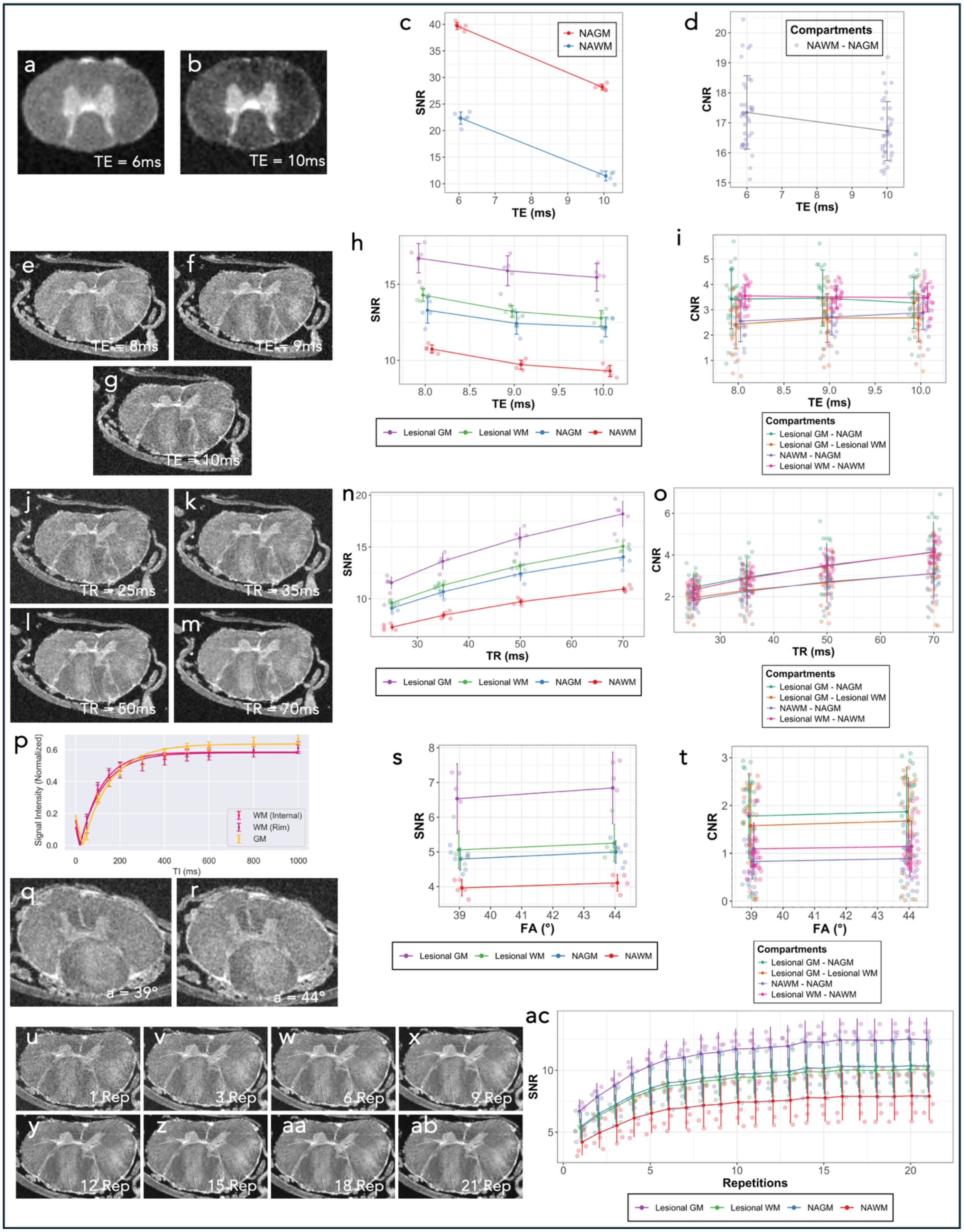
a
TE = 6ms
b
TE = 10ms
c
SNR
TE (ms)
NAGM
NAWM
d
CNR
TE (ms)
Compartments
NAWM - NAGM
e
TE = 8ms
f
TE = 9ms
g
TE = 10ms
h
SNR
TE (ms)
Lesional GM
Lesional WM
NAGM
NAWM
i
CNR
TE (ms)
Compartments
Lesional GM - NAGM
Lesional GM - Lesional WM
NAWM - NAGM
Lesional WM - NAWM
j
TR = 25ms
k
TR = 35ms
l
TR = 50ms
m
TR = 70ms
n
SNR
TR (ms)
Lesional GM
Lesional WM
NAGM
NAWM
o
CNR
TR (ms)
Compartments
Lesional GM - NAGM
Lesional GM - Lesional WM
NAWM - NAGM
Lesional WM - NAWM
p
Signal Intensity (Normalized)
TI (ms)
WM (Internal)
WM (Rim)
GM
q
a = 39°
r
a = 44°
s
SNR
FA (°)
Lesional GM
Lesional WM
NAGM
NAWM
t
CNR
FA (°)
Compartments
Lesional GM - NAGM
Lesional GM - Lesional WM
NAWM - NAGM
Lesional WM - NAWM
u
1 Rep
v
3 Rep
w
6 Rep
x
9 Rep
y
12 Rep
z
15 Rep
aa
18 Rep
ab
21 Rep
ac
SNR
Repetitions
Lesional GM
Lesional WM
NAGM
NAWM

Supplementary Figure 1. Optimization of T2*-weighted MRI for MR microscopy. (a-d) Representative MRI slices using echo times (TE) of 6ms (a) and (b), and corresponding signal-to-noise ratio (SNR, c) and contrast-to-noise ratio (CNR, d) in the marmoset spinal cord. (e-i) Representative MRI slices using echo times (TE) between 8-10ms (e-g), and corresponding SNR (h) and CNR (i) in the multiple sclerosis spinal cord. (j-o) Representative MRI slices using repetition times (TR) between 25-75ms (j-m), and corresponding SNR (n) and CNR (o) in the multiple sclerosis spinal cord. (p) T1 relaxation signal of a multiple sclerosis spinal cord. (q-t) Representative MRI slices using flip angles (a) between 39-44° (q-r), and corresponding SNR (s) and CNR (t) in the multiple sclerosis spinal cord. (u-ac) Representative MRI slices using numbers of repetitions (Rep) for each field-of-view between 1-21 (u-ab), and corresponding SNR (ac) in the multiple sclerosis spinal cord.

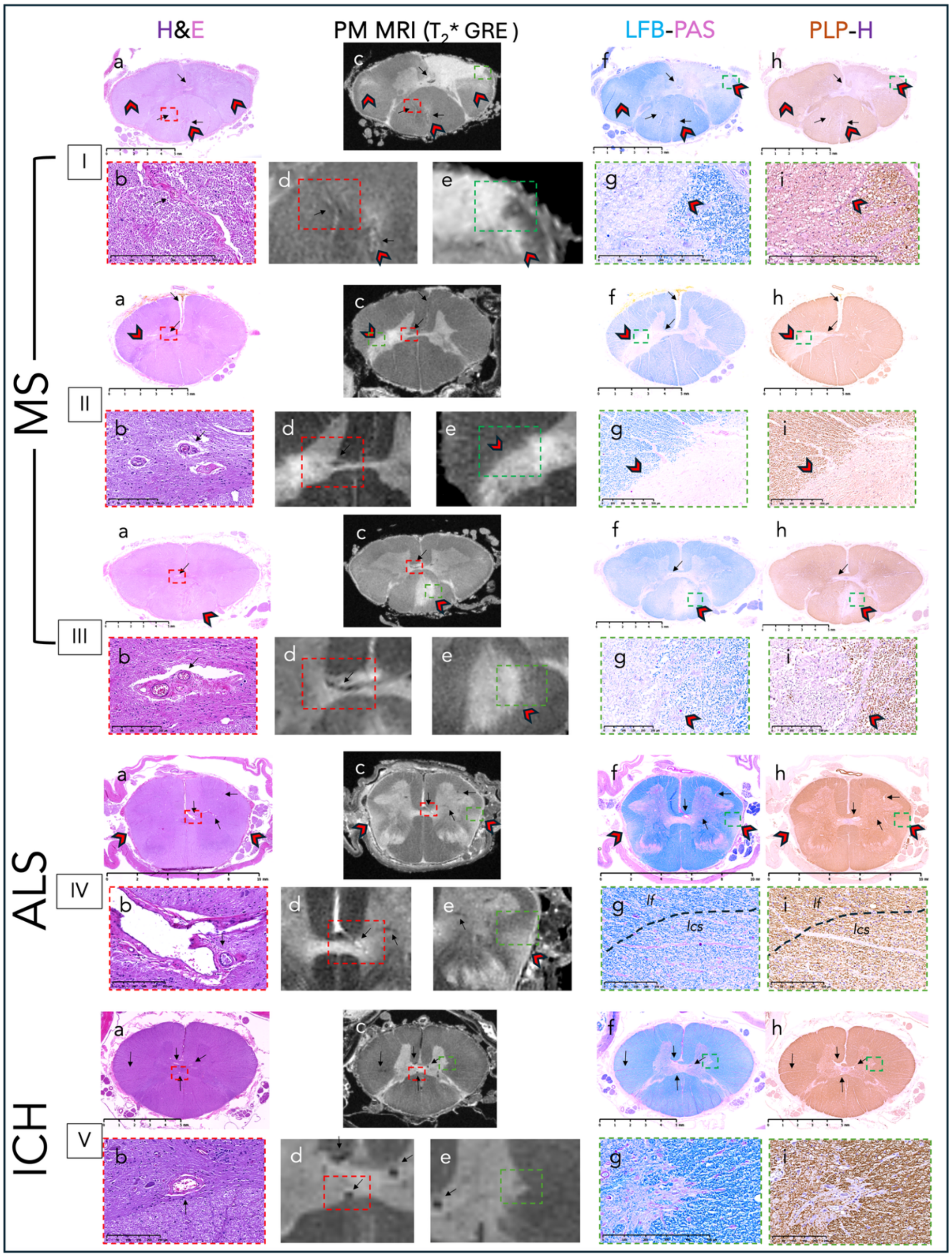

Supplementary Figure 2. Corresponding magnifications of T2*w postmortem MRI and histological sections from Fig. 5. (Ia-IIIh) Representative examples of MS SC lesions detected both in histology (I/II/IIIa-b & f-i) and on T2*w postmortem MRI (I/II/IIIc-e). (IVa-Vh) Representative histological (IV-Va-b & f-i) and postmortem MRI (IV/Vc-e) examples of ALS and ICH SC tissue. (I/II/III/IV/Va-b & f-i) Histological sections of whole transverse SC sections (I/II/III/IV/Va, f & h) and

magnified regions shown in red and green dashed rectangles (I/II/III/IV/V) stained with hematoxylin and eosin (I/II/III/IV/Va-b), Luxol fast blue combined with periodic acid-Schiff (I/II/III/IV/Vf-g), and myelin proteolipid protein (PLP) immunohistochemistry (I/II/III/IV/Vh-i) corresponding to respective postmortem MRI (I/II/III/IV/Vb-c). Corresponding axial T2*w postmortem MRI slices are shown in I/II/III/IV/Vc, and corresponding magnifications of red and green dashed rectangles are shown in /II/III/IV/Vd-e. Red arrowheads indicate the correspondence between features of demyelinating and neurodegenerative pathology on MRI and histology. Numerous demyelinated lesions were found in MS SC tissue (I/II/IIIc, e & f-i), while ALS SC exhibited bilateral degeneration of the lateral corticospinal tract (IVc, e & f-i). Black arrows indicate the correspondence between vessels seen both on MRI and histology. ALS: amyotrophic lateral sclerosis. MS: multiple sclerosis. ICH: intracranial hemorrhage. PM: postmortem. H&E: hematoxylin and eosin. LFB-PAS: Luxol fast blue combined with periodic acid-Schiff. PLP: myelin proteolipid protein. lcs: lateral corticospinal tract. lf: lateral funiculus.
