# Supplementary material for: A pipeline to characterize spinal cord pathology in neurological disorders combining magnetic resonance microscopy and histopathology
Source: Commun Med (Lond). 2026 Apr 22;6:370. doi: 10.1038/s43856-026-01577-8 (PMC13324037; doi:10.1038/s43856-026-01577-8)
Supplement: Supplementary file 3 — Description of Additional Supplementary files [file 43856_2026_1577_MOESM3_ESM.docx]

**Description of Additional Supplementary Files**

Supplementary Data File 1. MRI signal intensities and signal-to-noise-ratios in marmoset EAE and MS SC tissue incubated with different concentrations of Gad.

Supplementary Data File 2. MRI signal intensities and signal-to-noise-ratios in marmoset EAE SC tissue on T2*-weighted images with different echo times.

Supplementary Data File 3. MRI signal intensities and signal-to-noise-ratios in MS SC tissue on T2*-weighted images with different echo and repetition times.

Supplementary Data File 4. MRI signal intensities and signal-to-noise-ratios in MS SC tissue on T2*-weighted images with different flip angles.

Supplementary Data File 5. MRI signal intensities and signal-to-noise-ratios in MS SC tissue on T2*-weighted images with repetition numbers.
