# Supplementary material for: A pipeline to characterize spinal cord pathology in neurological disorders combining magnetic resonance microscopy and histopathology
Source: Commun Med (Lond). 2026 Apr 22;6:370. doi: 10.1038/s43856-026-01577-8 (PMC13324037; doi:10.1038/s43856-026-01577-8)
Supplement: Supplementary file 1 — Transparent Peer Review file [file 43856_2026_1577_MOESM1_ESM.pdf]

nature portfolio

# Peer Review File

# A pipeline to characterize spinal cord pathology in neurological disorders combining magnetic resonance microscopy and histopathology

Corresponding Author: Dr Maxime Donadieu

**This file contains all reviewer reports in order by version, followed by all author rebuttals in order by version.**

Version 0:

Reviewer comments:

Reviewer #1

(Remarks to the Author)
**Review for manuscript: A novel pipeline for characterizing spinal cord pathology in neurological disorders using magnetic resonance microscopy and histology**

This quite exciting work focuses on overcoming long-standing technical barriers in spinal cord (SC) imaging, which is in vivo particularly challenging because of the SC's small size, length, sensitivity to motion and anatomical location. Following the example of advances in brain blocks postmortem MRI the authors present a new postmortem pipeline that combines whole-SC MRI with targeted histological analysis. Their approach relies on a custom spinal cord holder, Gd-enhanced imaging to improve image quality, and a dedicated cutting system that enables MRI-guided tissue sampling. Together, these advances allow detailed, high-resolution analysis of the full spinal cord and open new possibilities for studying spinal cord diseases - especially because of the fact that this is to my knowledge the first time that SC pathology is imaged in such detail.
Overall, the work is mature, very well written and at a level of details that allows reproducibility. The methodological section is clearly structured, providing a proper guide in the details of the complex (yet needed) procedure and the addressed pitfalls. The imaging results effectively convey the full capabilities and potential of the proposed setup. The figures are well-thought and informative.
Based on my own experience with postmortem MRI acquisitions, I strongly support the publication of this manuscript. Below, I provide a number of minor comments aimed at further strengthening the contribution:

-----
**Lines 74-75**: The authors indicate: "Such scans have also helped better understand tissue microstructure and sources of brain MRI signal changes". 'Signal changes' is not a correct term in this context. I propose the phrasing: "Such experiments have also provided important insights into the relationships between tissue microstructure and the biophysical sources underlying brain MRI contrast."

**Line 84**: The current selection of references supporting the statement "Despite the previous development of robust methodological strategies for combined MRI and histological analysis of brain tissue" is limited and does not fully reflect recent methodological advances in this area. The authors are therefore encouraged to broaden the cited literature (substantially) to better capture contemporary developments. The same applies to some parts of the Discussion section, for example, Lines 280-282.
Specific suggestions are intentionally omitted to avoid any perception of selective citation; nevertheless, a range of relevant and well-established contributions can be readily identified in the recent literature.

**Lines 108-110**: Would be beneficial to mention why the fixation of the spine and subsequent dissection of the spinal cord is a better choice here than dissection fixation of the isolated cord on the first place.

**Lines 188-193**: Despite the usage of fiducial markers, the process could be benefited by the use of a methodology allowing for identification of the best histology-corresponding MR slice and a step of non-linear registration between the histology images and the corresponding MR slice. This, given that sectioning can cause deformation of the 5μm thick sections. Such technique is already used in relevant brain blocks research.

Please mention this within the limitations, in the discussion, providing also potential alternatives.

**Lines 255-257**: While the images presented in this work indeed represent a landmark achievement in postmortem spinal cord MRI, their translational potential remains limited. This is primarily due to (i) the absence of multimodal MRI contrasts—single-echo GRE provides excellent structural detail but limited additional biophysical information, (ii) the extremely high spatial resolution, which is currently unattainable in vivo, and (iii) the reliance on gadolinium-based contrast enhancement as an integral component of the approach.
Please integrate these points in the discussion.

**Lines 274-277**: Good points mentioned from the authors on ‘postmortem interval, associated diseases, and formalin fixation duration’. Please refer to previous work on brain samples that try to quantify these changes - can those effects be mitigated?

**Line 288**: “preliminary data suggest close correspondence between pre- and post-Gad incubation SC MRI”.
Are those data presented within the manuscript or elsewhere? If they are not available, there is no need to mention them, unless the authors intend to include a supplementary analysis, which in my view is not necessary at this stage. If they are available, please cite the source.
**Line 290: Please specify ‘images’: single-echo GRE that allow for identification of....**
**Line 293: Again here, as in the comment for Lines 255-257, as the imaging part is limited to a single structural contrast and after enhancement, i would not emphasize on the ‘understanding of MRI signal origins’ part. However, the work definitely allows for emphasis to the pathological elements. I propose to adjust the phasing.**

**Figure 5: Would be potentially helpful to see also the zoomed-in high resolution MRI contrast ROIs corresponding to the histology sections shown, either in the same figure or supplementary material (the latter probably better, for easier integration and not changing the main parts).**
**-----**

**Reviewer #2**

**(Remarks to the Author)**
**The central contribution of this work is the development of a custom tissue holder and container for postmortem MRI of the spinal cord. When inserted into a 9.4 T Bruker system, the setup enables imaging at an isotropic resolution of 75 µm with high SNR following gadolinium-based T1 shortening. In combination with a custom-built cutting rack, the authors demonstrate histological sectioning that corresponds closely to the MRI data. The overarching goal is to enable MRI-guided histology with high anatomical precision and, ultimately, to improve the interpretation of abnormal spinal cord MRI findings observed in clinical settings.**

**Overall, the paired hires MRI and histological images obtained using the proposed pipeline are technically impressive and clearly demonstrate careful experimental execution. However, the broader impact of the approach remains uncertain. The extremely long acquisition times and associated costs, the limited availability of 9.4 T systems, and most importantly the limited relevance of the resulting image contrast to clinical spinal cord MRI at lower field strengths substantially limit the translational value of the proposed pipeline.**

**More specifically, I have the following concerns:**

**1. The proposed imaging protocol provides only T1- and T2*-weighted contrast and does not address T2 contrast, which is the most clinically relevant contrast in human spinal cord MRI. Moreover, even the T1 contrast achieved here cannot be meaningfully related to in vivo MRI, as room-temperature imaging and the extensive preparation steps (including fixation and gadolinium immersion) introduce dramatic T1 shortening. Consequently, it appears very unlikely that this approach “bridges the gap between in vivo imaging and microscopic pathology,” as stated in the authors’ conclusions.**

**2. The manuscript states that the 3D-GRE sequences were optimized, but it is unclear with respect to which criteria. True optimization for SNR or CNR would require T1 mapping and simulation of the spoiled gradient-echo signal equation, which do not appear to have been performed. Therefore, it cannot be excluded that alternative parameter choices (e.g., flip angle and TR) could yield superior tissue contrast.**

**3. Acquisition time is a critical factor when assessing and comparing SNR. The manuscript reports an acquisition time of 45.9 minutes for a single average of the high-resolution post-Gd sequence; however, based on the reported parameters, one would expect a duration exceeding 100 minutes (≈ 0.045 × 200 × 730 s). Furthermore, the effective acquisition strategy is not presented in a clear or comprehensible manner. How many repetitions were required to achieve full coverage of the human spinal cord? While 12 repetitions should suffice to cover 60 cm (i.e., the full length of the tissue holder), Table 2 reports up to 21 repetitions. This discrepancy requires clarification.**

**4. The manuscript would benefit from a more detailed description of how the container was advanced between imaging segments. Was this performed manually? If so, was repositioning required every hour for 10 or more hours? This information is essential for assessing the practical feasibility and reproducibility of the proposed pipeline.**

Minor points:

5. In the introduction, the authors note that capturing the full length of the spinal cord is challenging due to long acquisition times; however, the proposed pipeline does not appear to mitigate this limitation.

6. The lettering is difficult to read in most figure and should be enlarged or improved for clarity.

7. Line 208: Local T2 variations should not affect signal intensity in a gradient-echo sequence with a short TE.

8. Line 274: The authors likely intend to refer to relaxation properties rather than magnetic properties, as magnetic properties do not influence T1 contrast.

Version 1:

Reviewer comments:

Reviewer #1

(Remarks to the Author)

Reviewer #2

(Remarks to the Author)
The authors have made considerable efforts to address all the raised points in a comprehensive and thoughtful manner. I have no additional comments.

Open Access This Peer Review File is licensed under a Creative Commons Attribution 4.0 International License, which permits use, sharing, adaptation, distribution and reproduction in any medium or format, as long as you give appropriate credit to the original author(s) and the source, provide a link to the Creative Commons license, and indicate if changes were made.
In cases where reviewers are anonymous, credit should be given to 'Anonymous Referee' and the source.
The images or other third party material in this Peer Review File are included in the article's Creative Commons license, unless indicated otherwise in a credit line to the material. If material is not included in the article's Creative Commons license and your intended use is not permitted by statutory regulation or exceeds the permitted use, you will need to obtain permission directly from the copyright holder.
To view a copy of this license, visit https://creativecommons.org/licenses/by/4.0/

**Reviewers' comments:**

**Reviewer #1 (Remarks to the Author):**

**Review for manuscript:** A novel pipeline for characterizing spinal cord pathology in neurological disorders using magnetic resonance microscopy and histology

**This quite exciting work focuses on overcoming long-standing technical barriers in spinal cord (SC) imaging, which is in vivo particularly challenging because of the SC's small size, length, sensitivity to motion and anatomical location. Following the example of advances in brain blocks postmortem MRI the authors present a new postmortem pipeline that combines whole-SC MRI with targeted histological analysis. Their approach relies on a custom spinal cord holder, Gd-enhanced imaging to improve image quality, and a dedicated cutting system that enables MRI-guided tissue sampling. Together, these advances allow detailed, high-resolution analysis of the full spinal cord and open new possibilities for studying spinal cord diseases -especially because of the fact that this is to my knowledge the first time that SC pathology is imaged in such detail.**

**Overall, the work is mature, very well written and at a level of details that allows reproducibility. The methodological section is clearly structured, providing a proper guide in the details of the complex (yet needed) procedure and the addressed pitfalls. The imaging results effectively convey the full capabilities and potential of the proposed setup. The figures are well-thought and informative.**

**Based on my own experience with postmortem MRI acquisitions, I strongly support the publication of this manuscript. Below, I provide a number of minor comments aimed at further strengthening the contribution:**

We thank the reviewer for the careful and thorough reading of our manuscript. We appreciate the positive assessment and the constructive nature of the comments provided. The reviewer's feedback has been helpful in strengthening the clarity and presentation of our work. We are grateful for the time and effort devoted to this review.

**Lines 74-75: The authors indicate: "Such scans have also helped better understand tissue microstructure and sources of brain MRI signal changes". 'Signal changes' is not a correct term in this context. I propose the phrasing: "Such experiments have also provided important insights into the**

**relationships between tissue microstructure and the biophysical sources underlying brain MRI contrast."**

Authors' response: We thank the reviewer for this suggestion. We adjusted our text accordingly.

**Line 84: The current selection of references supporting the statement "Despite the previous development of robust methodological strategies for combined MRI and histological analysis of brain tissue" is limited and does not fully reflect recent methodological advances in this area. The authors are therefore encouraged to broaden the cited literature (substantially) to better capture contemporary developments. The same applies to some parts of the Discussion section, for example, Lines 280-282.**

**Specific suggestions are intentionally omitted to avoid any perception of selective citation; nevertheless, a range of relevant and well-established contributions can be readily identified in the recent literature.**

Authors' response: Thank you for this suggestion. We added the citations shown below to our introduction.

24. Alkemade, A. et al. 7 Tesla MRI Followed by Histological 3D Reconstructions in Whole-Brain Specimens. Front Neuroanat 14, 536838 (2020).

25. Lahna, D. et al. Postmortem 7T MRI for guided histopathology and evaluation of cerebro-vascular disease. Journal of Neuropathology & Experimental Neurology 82, 57–70 (2023).

26. Gkotsoulias, D. G. et al. Bridging mesoscopic and microscopic scales in multiple sclero-sis: Postmortem brain block multi-contrast 9.4T MRI and histology quantification. STAR Protoc 6, 104203 (2025).

27. Singh, M. et al. Co-registration of In-Vivo Human MRI Brain Images to Postmortem Histo-logical Microscopic Images. Int J Imaging Syst Technol 18, 325–335 (2008).

28. Lahna, D. et al. Postmortem 7T MRI for guided histopathology and evaluation of cerebro-vascular disease. J Neuropathol Exp Neurol 82, 57–70 (2022).

29. Yilmazer-Hanke, D. et al. Histological correlates of postmortem ultra-high-resolution sin-gle-section MRI in cortical cerebral microinfarcts. acta neuropathol commun 8, 33 (2020).

30. Athalye, C. et al. Operationalizing postmortem pathology-MRI association studies in Alzheimer's disease and related disorders with MRI-guided histology sampling. Acta Neuropathol Commun 13, 120 (2025).

We also added the following citations in our discussion.

40. Boonstra, J. T., Michielse, S., Roebroeck, A., Temel, Y. & Jahanshahi, A. Dedicated con-tainer for postmortem human brain ultra-high field magnetic resonance imaging. Neu-roImage 235, 118010 (2021).

41. Dawe, R. J., Bennett, D. A., Schneider, J. A., Vasireddi, S. K. & Arfanakis, K. Postmortem MRI of Human Brain Hemispheres: T2 Relaxation Times during Formaldehyde Fixation. Magn Reson Med 61, 810–818 (2009).

42. Shatil, A. S., Matsuda, K. M. & Figley, C. R. A Method for Whole Brain Ex Vivo Magnetic Resonance Imaging with Minimal Susceptibility Artifacts. Front. Neurol. 7, (2016).

12. McDowell, A. R. et al. High-resolution quantitative MRI of multiple sclerosis spinal cord lesions. Magn Reson Med 87, 2914–2921 (2022).

13. Morris, S. et al. Advanced Magnetic Resonance Imaging Biomarkers of the Injured Spinal Cord: A Comparative Study of Imaging and Histology in Human Traumatic Spinal Cord In-jury. Journal of Neurotrauma 41, 1223–1239 (2024).

26. Gkotsoulias, D. G. et al. Bridging mesoscopic and microscopic scales in multiple sclero-sis: Postmortem brain block multi-contrast 9.4T MRI and histology quantification. STAR Protoc 6, 104203 (2025).

43. Galbusera, R. et al. Quantitative T1 is sensitive to cortical remyelination in multiple scle-rosis: A postmortem MRI study. Brain Pathol 35, e70010 (2025).

44. Neuhaus, D. et al. Comparative analysis of in situ and ex situ postmortem brain MRI: Evaluating volumetry, DTI, and relaxometry. Magnetic Resonance in Medicine 93, 213–227 (2025).

9. Calabrese, E. et al. Postmortem diffusion MRI of the entire human spinal cord at micro-scopic resolution.

**Lines 108-110: Would be beneficial to mention why the fixation of the spine and subsequent dissection of the spinal cord is a better choice here than dissection fixation of the isolated cord on the first place.**

Authors' response: Thank you for this suggestion. We believe that this is, indeed, an important methodological detail. We added the following sentence in that segment:

"Fixation of the spine before SC extraction was preferred over direct extraction at autopsy, as fixation-induced tissue stiffening facilitated laminectomy."

**Lines 188-193: Despite the usage of fiducial markers, the process could be benefited by the use of a methodology allowing for identification of the best histology-corresponding MR slice and a step of non-linear registration between the histology images and the corresponding MR slice. This, given that sectioning can cause deformation of the 5µm thick sections. Such technique is already used in relevant brain blocks research.**

**Please mention this within the limitations, in the discussion, providing also potential alternatives.**

Authors' response: We thank the reviewer for this suggestion. Indeed, in this study, we were able to visually identify the MRI slices that corresponded to our histological sections with high confidence, as demonstrated in Figure 5 and Supplementary Figure 2. We believe that the combined use of our custom-made spinal cord tissue holder, container, and cutting rack allowed for the acquisition of both axial MRI slices and histological sections that were perpendicular to the rostrocaudal spinal cord axis, as well as the use of fiducial markers during MRI acquisition, facilitated the identification of the best histology-corresponding MR slice. Nevertheless, as the reviewer suggested, this process could be potentially improved by a step of non-linear registration between the histology images and the corresponding MR slice, as previously performed in brain blocks, though this has not yet been validated in the spinal cord. We added this in our discussion as follows:

"Finally, in this study, we were able to visually identify the MRI slices corresponding to our histological sections. This was facilitated by the combined use of our custom-made SC tissue holder, container, and cutting rack that enabled the acquisition of both axial MRI slices and histological sections perpendicular

to the rostrocaudal SC axis, as well as by the use of fiducial markers during MRI acquisition. However, a more accurate identification of the optimal histology-corresponding MRI slice could potentially be achieved through the addition of non-linear registration to the pipeline, as previously implemented in postmortem brain blocks,[26,45–49] although such methods have not yet been validated in the SC."

**Lines 255-257: While the images presented in this work indeed represent a landmark achievement in postmortem spinal cord MRI, their translational potential remains limited. This is primarily due to (i) the absence of multimodal MRI contrasts—single-echo GRE provides excellent structural detail but limited additional biophysical information, (ii) the extremely high spatial resolution, which is currently unattainable in vivo, and (iii) the reliance on gadolinium-based contrast enhancement as an integral component of the approach.**

**Please integrate these points in the discussion.**

Authors' response: We agree with the reviewer regarding the limitations of our approach regarding clinical translation. The translational potential of our approach is related to its capability to validate in vivo structural MRI biomarkers, the discovery of previously undetected or under-detected pathology — which may be highly relevant in the spinal cord due to the current limitations of in vivo MRI — and understanding disease mechanisms as well as the cord's spatial cellular architecture. However, as the reviewer also mentioned, our approach does not allow for optimization of MRI sequences and contrasts that could be then applied in vivo, calibration of quantitative MRI metrics, and use of multimodal MRI contrasts in analogy to in vivo MRI. However, in postmortem MRI: i) relaxation times and diffusion properties are altered due to tissue fixation, postmortem interval, lower temperature, tissue dehydration and shrinkage, ii) susceptibility effects differ due to the lack of blood flow and oxygenation, and the exertion of stronger susceptibility effects by iron-rich microglia, myelin, and blood products, and iii) scanner-related and acquisition factors such as the use of ultra-high magnetic field (7T and above) and resolutions, and the use of custom coils and containers. All these factors, as well as the use of the gadolinium-based tissue preparation, generally limit the direct translation of postmortem MRI techniques to clinical application. We added part of this discussion in the study's limitations.

"In addition to several factors generally affecting postmortem MR signal,[43–46] the absence of multimodal MRI contrasts, the high spatial resolution (which is currently unattainable in vivo), and the reliance of our technique on gadolinium-based contrast enhancement hamper the clinical translation of our imaging approach. However, the translational potential of this method is related to its capability to: (i) validate in vivo structural MRI biomarkers (especially in the scenario in which both in vivo and postmortem images are available); (ii) discover previously undetected or under-detected pathology (which may be highly relevant in the spinal cord due to the current limitations of in vivo MRI); and (iii) potentially understand disease mechanisms and the cord's spatial biology."

**Lines 274-277: Good points mentioned from the authors on 'postmortem interval, associated diseases, and formalin fixation duration'. Please refer to previous work on brain samples that try to quantify these changes - can those effects be mitigated?**

Authors' response: We thank the reviewer for this suggestion. We cited previous work on brain samples and added mitigation strategies, as follows:

"However, postmortem interval, associated diseases, and formalin fixation duration can alter tissue relaxation times,[41,43–45] and a single protocol might not be ideal for all SC samples, although these factors could be partially mitigated by control of postmortem interval before fixation or by selection of SC specimens within a postmortem interval range, as well as standardizing fixation protocols and minimizing fixation times."

**Line 288: "preliminary data suggest close correspondence between pre- and post-Gad incubation SC MRI".**

**Are those data presented within the manuscript or elsewhere? If they are not available, there is no need to mention them, unless the authors intend to include a supplementary analysis, which in my view is not necessary at this stage. If they are available, please cite the source.**

Authors' response: Indeed, this close anatomical correspondence is visible in Figure 3e-h. We cited this figure in our text.

**Line 290: Please specify 'images': single-echo GRE that allow for identification of….**

Authors' response: We thank the reviewer for this suggestion. We adjusted our text accordingly.

**Line 293: Again here, as in the comment for Lines 255-257, as the imaging part is limited to a single structural contrast and after enhancement, I would not emphasize on the 'understanding of MRI signal origins' part. However, the work definitely allows for emphasis to the pathological elements. I propose to adjust the phasing.**

Authors' response: We agree with the reviewer that this statement may be misleading. We adjusted this sentence as follows:

"This approach provides a strong link between in vivo imaging and microscopic pathology, with the potential to deepen our understanding of the pathologic substrate of MRI abnormalities and their relationship with disease mechanisms in various neurological disorders affecting the SC."

**Figure 5: Would be potentially helpful to see also the zoomed-in high resolution MRI contrast ROIs corresponding to the histology sections shown, either in the same figure or supplementary material (the latter probably better, for easier integration and not changing the main parts).**

Authors' response: We thank the reviewer for this suggestion. We added Supplementary Figure 2 which includes the same examples with postmortem MRI magnifications of the corresponding areas highlighted in histology.

-----

**Reviewer #2 (Remarks to the Author):**

**The central contribution of this work is the development of a custom tissue holder and container for postmortem MRI of the spinal cord. When inserted into a 9.4 T Bruker system, the setup enables imaging at an isotropic resolution of 75 µm with high SNR following gadolinium-based T1 shortening. In combination with a custom-built cutting rack, the authors demonstrate histological sectioning that corresponds closely to the MRI data. The overarching goal is to enable MRI-guided histology with high anatomical precision and, ultimately, to improve the interpretation of abnormal spinal cord MRI findings observed in clinical settings.**

**Overall, the paired hires MRI and histological images obtained using the proposed pipeline are technically impressive and clearly demonstrate careful experimental execution. However, the broader impact of the approach remains uncertain. The extremely long acquisition times and associated costs, the limited availability of 9.4 T systems, and most importantly the limited relevance of the resulting image contrast to clinical spinal cord MRI at lower field strengths substantially limit the translational value of the proposed pipeline.**

We acknowledge the reviewer's thorough evaluation of the manuscript and the assessment provided. The comments were constructive and assisted in improving the clarity and presentation of the work.

**More specifically, I have the following concerns:**

**1. The proposed imaging protocol provides only T1- and T2*-weighted contrast and does not address T2 contrast, which is the most clinically relevant contrast in human spinal cord MRI. Moreover, even the T1 contrast achieved here cannot be meaningfully related to in vivo MRI, as room-temperature imaging and the extensive preparation steps (including fixation and gadolinium immersion) introduce dramatic T1 shortening. Consequently, it appears very unlikely that this approach "bridges the gap between in vivo imaging and microscopic pathology," as stated in the authors' conclusions.**

Authors' response: We thank the reviewer for this comment. We agree that the use of a high-field scanner, a high-resolution imaging protocol, and the tissue preparation by formalin fixation and immersion in gadolinium does not allow the comparison of our MR-images to those from a clinical setting. Indeed, our primary goal was to be able to identify spinal cord pathologies and target them for histopathology. Hence, the translational potential of our approach is related to its capability to: (i) validate in vivo structural MRI biomarkers (especially in the scenario in which both in vivo and postmortem images are available); (ii) discover previously undetected or under-detected pathology (which may be highly relevant in the spinal cord due to the current limitations of in vivo MRI); and (iii) potentially understand disease mechanisms and the cord's spatial biology. However, in addition to other factors limiting the translation of postmortem MRI approaches in clinical imaging, our approach does not allow for optimization of MRI sequences and contrasts that could be then applied in vivo (except in a relative sense), calibration of quantitative MRI metrics, and use of multimodal MRI contrasts in analogy to in vivo MRI. We did not intend to imply that the images and techniques applied in vivo and ex vivo are equivalent. In the end, we continue to think — and believe that our data demonstrate — that postmortem MRI, especially of spinal cord pathologies less clearly visible on in vivo MRI, will help us develop better in vivo imaging protocols in the future.

With respect to MRI contrasts, T1-, T2-, and T2*-weighted sequences (e.g. magnetization prepared rapid acquisition gradient echoes and related sequences, T2*-weighted single-/multi echo gradient echo sequences), as well as sequences such as short-tau inversion recovery (STIR) and magnetization transfer contrast, have all been shown to be sensitive for the detection of various spinal cord pathologies in vivo.

We added the following segment to our limitations:

"In addition to several factors generally affecting postmortem MR signal,[43–46] the absence of multimodal MRI contrasts, the high spatial resolution (which is currently unattainable in vivo), and the reliance of our technique on gadolinium-based contrast enhancement hamper the clinical translation of our imaging approach. However, the translational potential of this method is related to its capability to: (i) validate in vivo structural MRI biomarkers (especially in the scenario in which both in vivo and postmortem images are available); (ii) discover previously undetected or under-detected pathology (which may be highly relevant in the spinal cord due to the current limitations of in vivo MRI); and (iii) potentially understand disease mechanisms and the cord's spatial biology."

**2. The manuscript states that the 3D-GRE sequences were optimized, but it is unclear with respect to which criteria. True optimization for SNR or CNR would require T1 mapping and simulation of the spoiled gradient-echo signal equation, which do not appear to have been performed. Therefore, it cannot be excluded that alternative parameter choices (e.g., flip angle and TR) could yield superior tissue contrast.**

Authors' response: We acknowledge that the optimization parameters of the sequence used for MR microscopy are indeed important. To select our T2*-weighted MRI sequence, we experimented with basic sequence parameters such as the echo time, the repetition time, the flip angle, and the repetitions acquired for each FOV. First, we observed increased SNR and marginally increased CNR using a TE of 6ms compared to 10ms in the marmoset spinal cord. In a similar experiment of multiple sclerosis spinal cord tissue using TE of 8-10ms (lower TE were not feasible at 100 μm isotropic resolution due to MR system limitations), we observed slightly increased SNR for spinal cord compartments between 8ms and 10ms (normal-appearing and lesional WM: $p < 0.01$; normal-appearing and lesional GM: $p < 0.05$). Hence, for MR microscopy at 75 μm isotropic resolution, we selected the lowest feasible TE value of 9ms. Next, using a TE of 9ms, we assessed the effect of TR values between 25-70ms. At 100 μm isotropic resolution, we observed a significantly higher SNR with higher TR values (all $p < 0.001$), but a smaller increase in CNR using a TR of 70ms compared to 50ms (normal-appearing WM/GM to lesional WM/GM: $p < 0.5$; normal-appearing WM to normal-appearing GM: not significant; lesional WM to lesional GM: not significant). Since longer TR values came at a cost of longer acquisition times, we selected a TR of 45ms for MR microscopy at 75 μm isotropic resolution. To calculate the optimal flip angle, we acquired, before gadolinium-based tissue preparation, magnetization-prepared rapid acquisition gradient echo images (300 μm isotropic resolution, TR=4000ms, TE=1ms, flip angle 8°) at multiple TI: 50ms, 100, 150ms, 200ms, 300ms, 400ms, 500ms, 600ms, 800ms, 1000ms) and generated spinal cord T1 maps. We calculated a T1 value of 123.8 ± 1.3 at the spinal cord gray matter, corresponding to a flip angle ≈ 46° for TR = 45 ms using the Ernst equation, while lesions had variably longer T1 values. To obtain maximal signal from the spinal cord gray matter and lesions, we subsequently experimented with flip angles slightly lower than 46° and observed slightly higher SNR and CNR at 44° compared to 39° (difference not statistically significant). Finally, we calculated the number of repetitions required in each field-of-view for SNR maximization. Since there was minimal SNR gain after 12 repetitions at 75 μm isotropic resolution, we selected this as the minimum number of repetitions for each field-of-view in our experiments. We added this discussion to our main text and a supplementary figure as follows:

"Optimized imaging protocols are detailed in Table 2, and the sequence optimization process is described in the Supplementary Material and Supplementary Fig. 1."

## MRI sequence optimization

"To optimize our T2*-weighted MRI sequence, we experimented with basic sequence parameters such as the echo time, the repetition time, the flip angle, and the repetitions acquired for each FOV (Supplementary Fig. 1). First, we observed increased SNR and marginally increased CNR using a TE of 6ms compared to 10ms in the marmoset spinal cord. In a similar experiment of multiple sclerosis spinal cord tissue using TE of 8-10ms (lower TE were not feasible at 100 μm isotropic resolution due to MR system limitations), we observed slightly increased SNR for spinal cord compartments between 8ms and 10ms (normal-appearing and lesional WM: $p < 0.01$; normal-appearing and lesional GM: $p < 0.05$). Hence, for MR microscopy at 75 μm isotropic resolution, we selected the lowest feasible TE value of 9ms. Next, using a TE of 9ms, we assessed the effect of TR values between 25-70 ms. At 100 μm isotropic resolution, we observed a significantly higher SNR with higher TR values (all $p < 0.001$), but a smaller increase in CNR using a TR of 70ms compared to 50ms (normal-appearing WM/GM to lesional WM/GM: $p < 0.5$; normal-appearing WM to normal-appearing GM: not significant; lesional WM to lesional GM: not significant). Since longer TR values came at a cost of longer acquisition times, we selected a TR of 45ms for MR microscopy at 75 μm isotropic resolution. To calculate the optimal flip angle, we acquired, before gadolinium-based tissue preparation, magnetization-prepared rapid acquisition gradient echo images (300 μm isotropic resolution, TR=4000ms, TE=1ms, flip angle 8°) at multiple TI: 50ms, 100, 150ms, 200ms, 300ms, 400ms, 500ms, 600ms, 800ms, 1000ms) and generated spinal cord T1 maps. We calculated a T1 value of 123.8 ± 1.3 at the spinal cord gray matter, corresponding to a flip angle ≈ 46° for TR = 45 ms using the Ernst equation, while lesions had variably longer T1 values. To obtain maximum signal from the spinal cord gray matter and lesions, we subsequently experimented with flip angles slightly lower than 46° and observed slightly higher SNR and CNR at 44° compared to 39° (difference not statistically significant). Finally, we calculated the number of repetitions required in each field-of-view for SNR maximization. Since there was minimal SNR gain after 12 repetitions at 75 μm isotropic resolution, we selected this as the minimum number of repetitions for each field-of-view in our experiments."

**3. Acquisition time is a critical factor when assessing and comparing SNR. The manuscript reports an acquisition time of 45.9 minutes for a single average of the high-resolution post-Gd sequence; however, based on the reported parameters, one would expect a duration exceeding 100 minutes (≈ 0.045 × 200 × 730 s). Furthermore, the effective acquisition strategy is not presented in a clear or comprehensible manner. How many repetitions were required to achieve full coverage of the human spinal cord? While 12 repetitions should suffice to cover 60 cm (i.e., the full length of the tissue holder), Table 2 reports up to 21 repetitions. This discrepancy requires clarification.**

Authors' response: We acknowledge this comment. However, this is the way we calculated the acquisition time:

Our given matrix is 200x306x730 (AP x RL x SI). For a 3D GRE sequence, only phase encoding affects scan time and not frequency encoding. By convention, the largest matrix dimension is almost always frequency encoding (to minimize echo spacing and bandwidth penalties).

Now we apply the acquisition time calculation equation as follow:

Acquisition Time (AT)= TR (in sec) x Phase encoding 1 x Phase encoding 2 x number of average/repetition (NSA)

For our T2* 3D-GRE it will be AT =0.045 x 200 x 306 x 1 (for 1 average/repetition)

So, AT = 2754 s = **45.9 min** (for 1 average/repetition)

As MRI acquisition matrices are usually presented in the form of RL x AP x SI, while our acquisition matrix in Table 2 is in the form of **AP x RL x SI,** we believe that this might be confusing for the reader and adjusted our Table 2 accordingly.

We hope that the paragraph added for comment #2 includes enough detail regarding our acquisition strategy.

To clear any confusion about the repetition number, 12 is the minimum number of repetitions acquired per SC segment (the gain in SNR was minimal after 12 repetitions at 75 µm isotropic resolution). To cover the entire spinal cord (~43-45 cm), up to 9 segments were necessary. We clarified those points in the text as follows:

"To achieve full cord coverage, the SC was scanned in multiple segments (12 repetitions minimum per segment; up to 9 segments required to scan the entire SC), each with a 5.5-cm superior-inferior FOV and a 0.5-cm overlap between subsequent segments to avoid unscanned SC areas and facilitate consecutive image stitching".

**4. The manuscript would benefit from a more detailed description of how the container was advanced between imaging segments. Was this performed manually? If so, was repositioning required every hour for 10 or more hours? This information is essential for assessing the practical feasibility and reproducibility of the proposed pipeline.**

Authors' response: We acknowledge that point and want to remove any confusion on how the SC was moved between acquisitions. As stated in comment #2 and #3 answers, each SC was entirely covered in up to 9 segments. Each segment was scanned with a minimum of 12 repetitions; thus, the container had to be roughly moved every 9.2 hours to maintain continuous scanning. We added details in the text as follows:

"The container was advanced in the scanner using a ruler after MRI acquisition of each SC segment (roughly every 9.2 hours), and the scan was repeated until the entire SC was scanned".

**Minor points:**

**5. In the introduction, the authors note that capturing the full length of the spinal cord is challenging due to long acquisition times; however, the proposed pipeline does not appear to mitigate this limitation.**

Authors' response: We acknowledge the reviewer's point and made the sentence in the introduction simpler and more straightforward as follows:

"Breakthroughs in gradient design, boosting both maximum strength and slew rates, have allowed for sub-100 µm isotropic resolution in postmortem MRI despite long acquisition times and $B_0$ inhomogeneities along the field-of-view (FOV)."

**6. The lettering is difficult to read in most figure and should be enlarged or improved for clarity.**

Authors' response: Thank you for bringing up this point. We increased the lettering font size in all figures.

**7. Line 208: Local T2 variations should not affect signal intensity in a gradient-echo sequence with a short TE.**

Authors' response: We acknowledge the reviewer's point. We removed this from our text.

**8. Line 274: The authors likely intend to refer to relaxation properties rather than magnetic properties, as magnetic properties do not influence T1 contrast.**

Authors' response: Thank you for this comment. We were indeed referring to relaxation properties. Hence, we adjusted our text accordingly.
